# Allostatic Changes in the cAMP System Drive Opioid-Induced Adaptation in Striatal Dopamine Signaling

**DOI:** 10.1016/j.celrep.2019.09.034

**Published:** 2019-10-22

**Authors:** Brian S. Muntean, Maria T. Dao, Kirill A. Martemyanov

**Affiliations:** 1 Department of Neuroscience, The Scripps Research Institute, Jupiter, FL 33458, USA; 2 Lead Contact

## Abstract

Opioids are powerful addictive agents that alter dopaminergic influence on reward signaling in medium spiny neurons (MSNs) of the nucleus accumbens. Repeated opioid exposure triggers adaptive changes, shifting reward valuation to the allostatic state underlying tolerance. However, the cellular substrates and molecular logic underlying such allostatic changes are not well understood. Here, we report that the plasticity of dopamine-induced cyclic AMP (cAMP) signaling in MSNs serves as a cellular substrate for drug-induced allostatic adjustments. By recording cAMP responses to optically evoked dopamine in brain slices from mice subjected to various opioid exposure paradigms, we define profound neuronal-type-specific adaptations. We find that opioid exposure pivots the initial hyper-responsiveness of D1-MSNs toward D2-MSN dominance as dependence escalates. Presynaptic dopamine transporters and postsynaptic phosphodiesterases critically enable cell-specific adjustments of cAMP that control the balance between opponent D1-MSN and D2-MSN channels. We propose a quantitative model of opioid-induced allostatic adjustments in cAMP signal strength that balances circuit activity.

## In Brief

Muntean et al. examine how opioid exposure influences cyclic AMP (cAMP) responses to dopamine in striatal medium spiny neurons (MSNs). They describe allostatic adaptations in the processing of dopaminergic signals by D1-MSN and D2-MSN populations as opioid administration progresses from acute exposure to chronic use, and they define molecular elements contributing to the process.

## Graphical Abstract

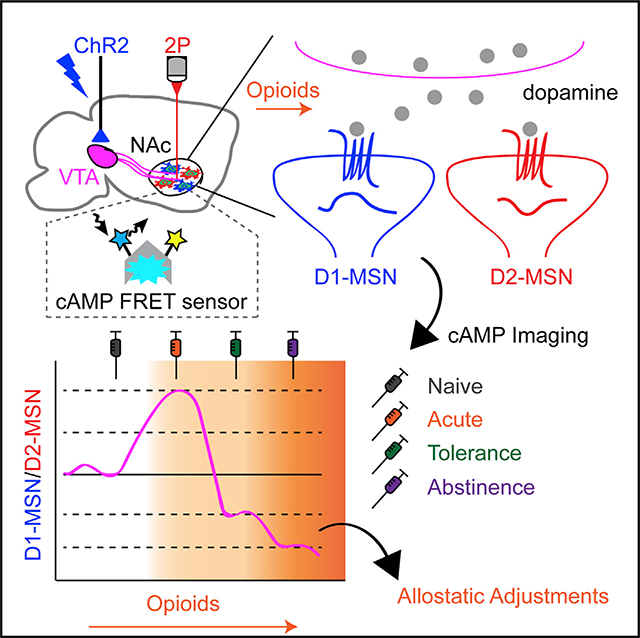

## INTRODUCTION

The anatomical connectome of the mammalian brain provides the infrastructure for synaptic communication, which underlies functional neuronal circuits instructing action selection. A remarkable feature of neural systems is the boundless adaptable plasticity in response to input stimuli, allowing response adjustments based on experience while maintaining homeostatic integrity (Josselyn et al., 2017). This property is prominently featured in the mesolimbic dopamine system (Reynolds and Wickens, 2002), which constitutes the core requirements for the refinement of motor sequences (Graybiel, 2000) and reward valuation (Lobo and Nestler, 2011).

The basal ganglia contain two prominent dopaminergic inputs: dopamine neurons from the midbrain substantia nigra pars compacta (SNc) and ventral tegmental area (VTA) project to the striatal caudate putamen (CPu) and nucleus accumbens (NAc), respectively (Gerfen, 1992). Dopamine processing in the CPu is required for action initiation/selection, and disorders such as Parkinson’s disease are characterized by the loss of SNc projections and profound movement dysfunctions (Graybiel, 2000). In contrast, reward signals are encoded through phasic patterns of dopamine release (Tsai et al., 2009), processed by the NAc, to calculate both positive reinforcement and noxious reactions (Schultz, 2013). In this scenario, dopamine serves as a biasing factor in the selection of appetitive behaviors by providing a temporal reward prediction error (RPE) (Schultz, 2016). The RPE embodies the difference between the actual stimuli-induced reward and the estimated reward value forecast based on prior experience to steer future behavior toward reward acquisition (Schultz et al., 2017). This process is most directly observed during exposure to drugs of abuse (i.e., cocaine, amphetamines, opioids) (Covey et al., 2014), which dramatically enhance dopamine levels (Di Chiara and Imperato, 1988) to induce euphoria during a brief window followed by a dysphoric state upon withdrawal of drug use (Pothos et al., 1991; Rossetti et al., 1992a, 1992b; Tjon et al., 1994). Changes in the set point of dopamine and reward valuation during this process are thought to follow principles of allostasis (George et al., 2012). This theory argues that a repeated cyclical elevation of dopamine levels triggers an adjustment of reward valuation to a new allostatic set point elevating the reward threshold, driving persistent drug-seeking behavior in an effort to overcome the dysphoria associated with the addictive state (Koob and Le Moal, 2001). However, the molecular and cellular substrates driving the allostatic adaptations in the reward circuit, as well as mechanisms of this process, are unknown. Among drugs of abuse, opioids occupy a special place, carrying the highest addictive liability (Volkow et al., 2019), which is thought to be due to the strength of the allostatic adaptations that they exert (Gradin et al., 2014; Martin-Soelch et al., 2001). Indeed, opioid exposure induces adaptations clearly discernable by behavioral reactions such as strong euphoria, marked tolerance, and intense withdrawal reactions (Donroe and Tetrault, 2017). Opioids intervene at multiple points in the reward circuitry, exerting synergistic effects on the dopaminergic system (Di Chiara and Imperato, 1988; Pothos et al., 1991). Thus, opioids present a convenient model for understanding how exposure to addictive drugs affects the processing of dopaminergic signals to produce allostatic shifts in reward valuation.

The NAc is composed of ~95% medium spiny neurons (MSNs), which are the key element in processing dopamine signals. Two major subpopulations of MSNs feature distinct transcriptional landscapes, most prominently delineated by differential expressions of either the dopamine D1 receptor (D1-MSNs) or the dopamine D2 receptor (D2-MSNs) (Gerfen et al., 1990; Gokce et al., 2016). These populations control reward valuation in opposing fashions: D1-MSNs are involved during behaviors related to rewards, whereas D2-MSNs are thought to be integrated with aversive stimuli (Hikida et al., 2010; Kravitz et al., 2012; Tai et al., 2012). Prevalent models posit that the bidirectional control of MSN populations by dopamine, which generally has stimulatory effects on D1-MSNs and inhibitory effects on D2-MSNs, dictates behavioral outcomes (Tecuapetla et al., 2014; Yttri and Dudman, 2016). It is the synchronized responses of MSNs to changes in dopaminergic inputs that are thought to program goal-directed activity (Tobler et al., 2005). Yet, the nature of these computations and the cellular mechanisms that underlie the parallel processing of dopamine signals as well as their plasticity has been difficult to ascertain, mainly due to the lack of direct and immediate changes in the electrical properties of MSNs to dopamine (Cepeda et al., 1998; Malenka and Kocsis, 1988; Nicola and Malenka, 1998; Shen et al., 2008; Zheng et al., 1999). To tackle this issue, genetically encoded reporter-based approaches are being increasingly adopted. Thus far, these efforts included measuring either dopamine itself as it is released synaptically onto MSNs utilizing engineered dopamine receptors (Patriarchi et al., 2018; Sun et al., 2018) or measuring the overall activity of MSNs by calcium indicators (i.e., GCaMP) (Calipari et al., 2016; Cui et al., 2014a, 2013; Tecuapetla et al., 2014). While providing important information on dopamine actions and the roles of MSN populations in behavioral actions, these studies do not target the information-decoding process performed by D1-MSNs and D2-MSNs, which requires looking at events triggered by dopamine receptor activation. Such information is powerfully provided by monitoring the dopamine-driven activation of ectopically overexpressed G protein inwardly rectifying K+ channel (GIRK) channels, directly activated by Gβγ subunits released from Gi/o proteins (Marcott et al., 2014). However, this approach is limited to decoding dopamine actions only in D2-MSNs while monitoring an event not physiologically engaged in by MSNs.

The major physiological and direct cellular consequence of dopamine action on both MSN populations is the positive and negative influence on cyclic AMP (cAMP) production mediated by the D1 and D2 dopamine receptors, respectively (Lobo and Nestler, 2011). There is considerable evidence pointing to the role of dopamine-triggered cAMP alterations in programing reward valuation and in the actions of addictive drugs (Di Chiara and Imperato, 1988; Self et al., 1996; Svenningsson et al., 2005; Volkow et al., 2009). This is further supported by earlier work implicating cAMP changes in the NAc in reward-related behaviors (Carlezon et al., 1998; Self et al., 1998), yet the mechanisms of these effects are still being uncovered (Nestler, 2012). At the cellular level, cAMP orchestrates a number of physiological responses including changes in excitability, synaptic plasticity, and gene expression through a variety of its effector ion channels, as well as transcription factors (Kandel, 2012). Interestingly, the cAMP system is also subject to elaborate homeostatic adaptation that affects the processing of neuromodulatory G protein coupled receptor (GPCR) responses (Fan et al., 2009; Terwilliger et al., 1991; Watts and Neve, 2005). Thus, cAMP dynamics serve as a powerful proxy for monitoring the neuromodulatory effects on neural response generation and accompanying computations performed by the circuit.

In this study, we utilized the recently developed *in vivo* tool offered by the cAMP-encoded reporter (*CAMPER*) mice (Muntean et al., 2018) to interrogate the plasticity of the mesolimbic dopamine system underlying the development of adaptive changes that drive opioid addiction in an intact reward circuit with cell-specific precision. We identified key molecular components that shape the plasticity of cAMP signaling and delineated key adaptations in the circuit during coordinated activity between neurons that calculate reward value. On the basis of our findings, we propose a multi-parametric model by which dopamine signal integration is allostatically tuned following exposure to opioids.

## RESULTS

### A Single Acute Opioid Exposure Differentially Alters the Processing of Dopamine Signals in Subpopulations of Striatal Neurons

**Figure 1.**
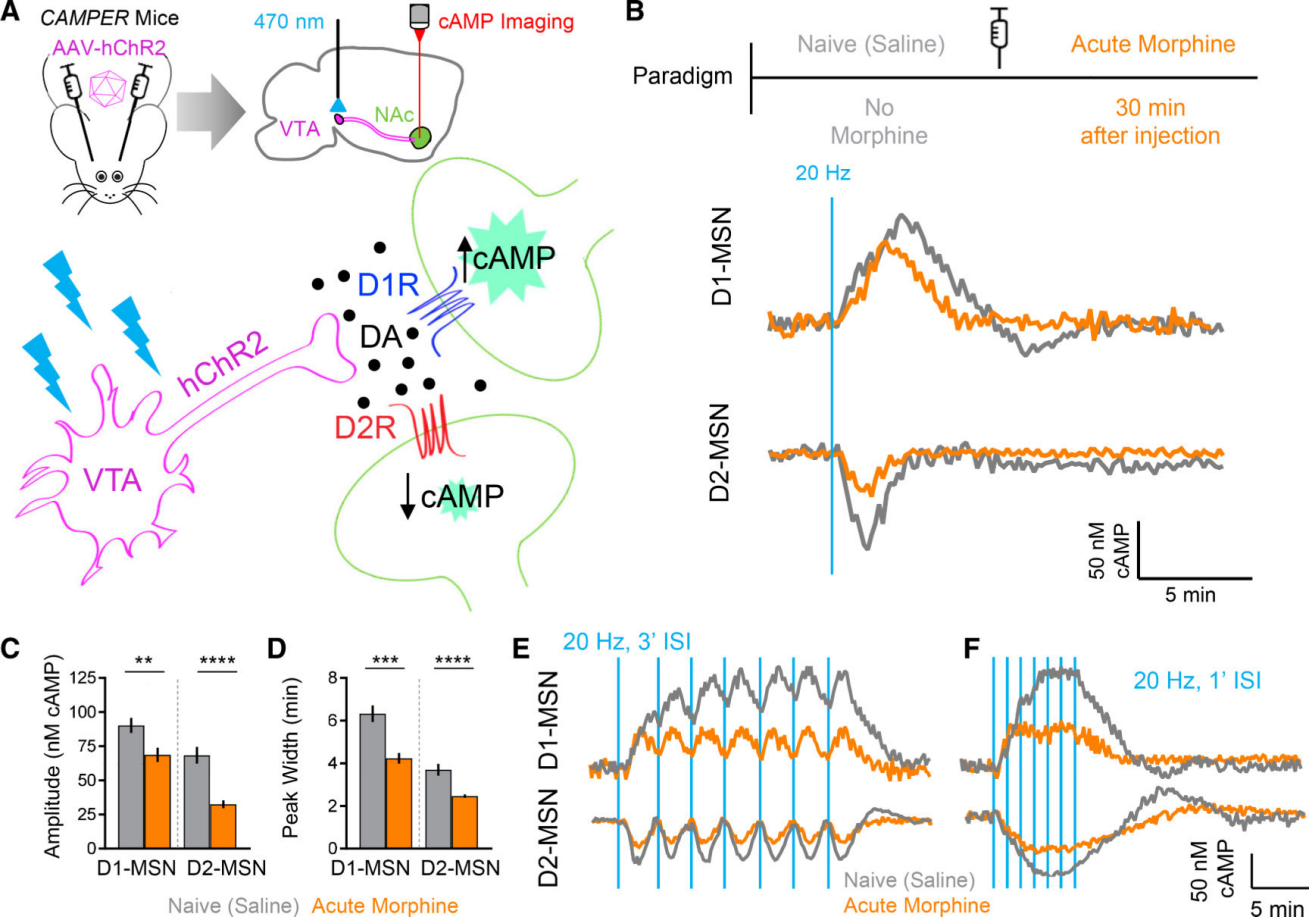
A Single Acute Exposure to Morphine Induces Changes in cAMP Signaling in the NAc (A) Experimental approach utilizing *CAMPER* brain slices. (B) cAMP responses recorded following optical stimulation. (C) Max amplitude cAMP change. Naive D1-MSN 90.3 ± 5.5 nM cAMP (n = 51 neurons; 7 mice) versus acute D1-MSN 68.7 ± 5.2 nM cAMP (n = 41 neurons; 6 mice); t test p = 0.0066, Kolmogorov-Smirnov (KS) D = 0.3544. Naive D2-MSN 68.5 ± 6.1 nM cAMP (n = 47 neurons; 6 mice) versus acute D2-MSN 32.5 ± 2.9 nM cAMP (n = 40 neurons; 5 mice); t test p < 0.0001, KS D = 0.516. (D) Peak width quantification. Naive D1-MSN 6.3 ± 0.39 min (n = 51 neurons; 7 mice) versus acute D1-MSN 4.2 ± 0.25 min (n = 41 neurons; 6 mice); t test p = 0.0003, KS D = 0.4414. Naive D2-MSN 3.7 ± 0.27 min (n = 47 neurons; 6 mice) versus acute D2-MSN 2.5 ± 0.08 min (n = 40 neurons; 5 mice); t test p < 0.0001, KS D = 0.5883. (E) cAMP responses from 20-Hz stimulation at 3′ inter-stimulation intervals (ISIs). (F) cAMP responses from 20-Hz stimulation at 1′ ISIs.

To begin probing the plasticity of dopamine signaling in the mesolimbic reward circuit and adaptations triggered by addictive morphine, we utilized an optogenetic strategy for inducing dopamine release from the VTA while simultaneously recording dopamine actions on D1-MSNs and D2-MSNs by monitoring real-time changes in intracellular cAMP (Figure 1A). This approach utilizes the recently developed *CAMPER* mouse model, which has been calibrated to report absolute cAMP values with nanomolar resolution (Muntean et al., 2018). In particular, this strategy utilizes a cocktail of receptor antagonists validated to isolate dopaminergic inputs (Marcott et al., 2014; Muntean et al., 2018). Thus, in acute brain slice preparations, we first examined adaptive changes induced by a single dose of morphine, administered to animals 30 min prior to the experiment, while comparing the effects to drug-naive mice (Figure 1B). Notably, this protocol induced profound cell-specific changes in baseline cAMP levels, such that the acute treatment nearly doubled the cAMP in D1-MSNs, whereas the D2-MSNs were unaffected (Figure S1A). Interestingly, acute opioid exposure massively altered the processing of dopamine inputs triggered by optogenetic stimulation in both populations of MSNs (Figure 1B). We detected a significant reduction in both response amplitudes and durations in D1-MSNs as well as D2-MSNs in morphine-treated mice (Figures 1C and 1D).

To understand the implication of these alterations on circuit properties, we next examined the integration of phasic dopamine transients, which are considered a hallmark of reward reinforcement calculations by the dopamine system (Di Chiara and Imperato, 1988; Phillips et al., 2003). The shape of the response is expected to significantly contribute to the integration of circuit activity, which we modeled by varying the intervals between individual stimulation bouts delivered while analyzing the response summation in both naive and morphine-exposed mice. Delivery of repeated bouts mimicking endogenous VTA phasic firing frequency of ~20 Hz (Juarez and Han, 2016) spaced at 3-min inter-stimulation intervals (ISIs) resulted in a response summation in drug-naive D1-MSNs (Figure 1E). In contrast, the drug-naive D2-MSN population exhibited no evidence of a summation, and the individual peaks were fully resolved. Morphine exposure markedly inhibited the summation of the response by the D1-MSNs, consistent with the increase in the temporal resolution of elemental responses under these conditions (Figure 1E). To investigate the effect of morphine on the response summation properties of D2-MSNs, we reduced the ISI to 1 min. Indeed, this produced a fusion of the responses into a single tetanic wave (Figure 1F). The response was also summed following morphine exposure; however, its amplitude was diminished, and its recovery was clearly compromised. These effects were even more pronounced at a shorter ISI of 0.5 min (Figures S1B and S1C). Taken together, these results demonstrate that acute opioid exposure results in considerable alterations in the dopaminergic responses of striatal MSNs. This includes characteristic subtype-selective effects, compromising their sensitivity and kinetic properties, which significantly influence the integration of phasic dopamine signals from the VTA.

### Chronic Opioid Exposure Reveals Pathway-Selective Plasticity of Dopaminergic VTA-NAc Signaling

**Figure 2.**
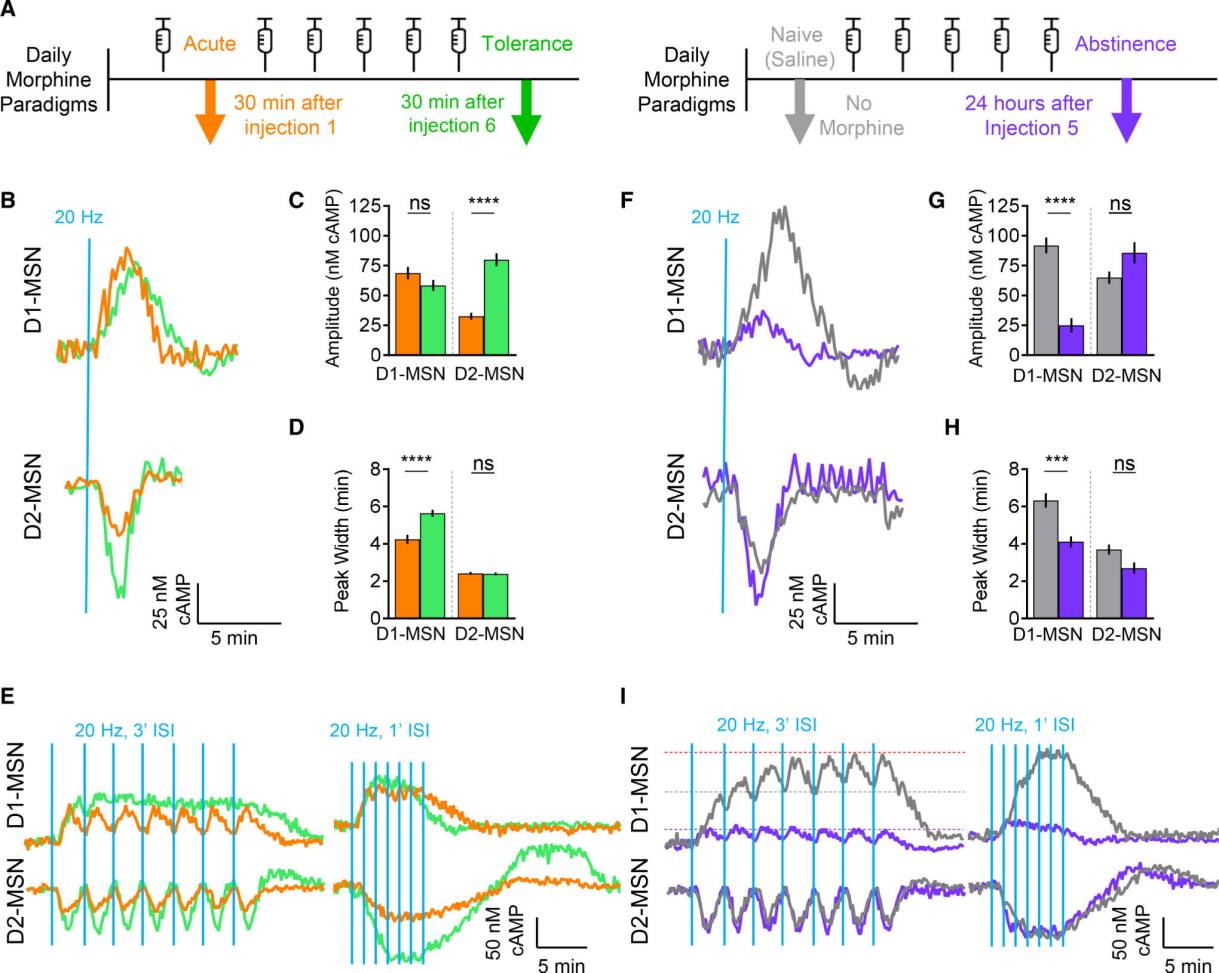
Morphine Exposure Induces Allostatic Changes in cAMP Signaling (A) Morphine administration paradigms. (B) cAMP response from optical stimulation. (C) Max amplitude cAMP change. Acute D1-MSN 68.7 ± 5.2 nM cAMP (n = 41 neurons; 6 mice) versus tolerance D1-MSN 58 ± 4.5 nM cAMP (n = 39 neurons; 4 mice); t test p = 0.275, KS D = 0.2226. Acute D2-MSN 32.5 ± 2.9 nM cAMP (n = 40 neurons; 5 mice) versus tolerance D2-MSN 80 ± 5.4 nM cAMP (n = 46 neurons; 5 mice); t test p < 0.0001, KS D = 0.7261. (D) Response duration. Acute D1-MSN 4.2 ± 0.25 min (n = 41 neurons; 6 mice) versus tolerance D1-MSN 5.6 ± 0.19 min (n = 39 neurons; 4 mice); t test p < 0.0001, KS D = 0.6048. Acute D2-MSN 2.5 ± 0.08 min (n = 40 neurons; 5 mice) versus tolerance D2-MSN 2.4 ± 0.07 min (n = 46 neurons; 5 mice); t test p = 0.5208, KS D = 0.1761. (E) cAMP response from optical stimulation at 3′ or 1′ ISIs. (F) cAMP response from optical stimulation. (G) Max amplitude cAMP change. Naive D1-MSN 90.3 ± 5.5 nM cAMP (n = 51 neurons; 7 mice) versus abstinence D1-MSN 29.1 ± 4.9 nM cAMP (n = 35 neurons; 6 mice); t test p < 0.0001, KS D = 0.702. Naive D2-MSN 68.5 ± 6.1 nM cAMP (n = 47 neurons; 6 mice) versus abstinence D2-MSN 84.5 ± 7.2 nM cAMP (n = 38 neurons; 5 mice); t test p = 0.3572, KS D = 0.2021. (H) Response duration. Naive D1-MSN 6.3 ± 0.39 min (n = 51 neurons; 7 mice) versus abstinence D1-MSN 4.1 ± 0.29 min (n = 35 neurons; 6 mice); t test p = 0.0009, KS D = 0.4314. Naive D2-MSN 3.7 ± 0.27 min (n = 47 neurons; 6 mice) versus abstinence D2-MSN 2.7 ± 0.30 min (n = 38 neurons; 5 mice); t test p = 0.0641, KS D = 0.2861. (I) cAMP response from optical stimulation at 3′ or 1′ ISIs. Gray/purple line indicates initial response peak. Red line indicates max response.

In parallel with the previous experiments, we designed our study to simultaneously examine long-term adaptations in processing dopamine signals by D1- and D2-MSNs induced by chronic exposure to opioids. Using the same approach, we compared four paradigms of opioid exposure, which included the previously described naive state (saline) and acute opioid exposure (Figure S2A). In addition, we studied adaptations underlying the development of tolerance, which have been observed following several sessions of intermittent opioid exposure, as well as the final paradigm of abstinence of opioids following repeated exposures (Madia et al., 2009; Posa et al., 2016) (Figure 2A). In the tolerance protocol, morphine exposure was extended to a total of six daily injections, where acute brain slices were prepared 30 min after the final injection. In order to extract alterations specifically induced by repeated exposure, results were compared directly to those following the single injection of morphine (Figure 1). We found that relative to acute exposure, upon chronic treatment, D1-MSNs exhibited significantly lower basal cAMP levels (Figure S2B), amid an unaltered response amplitude (Figures 2B and 2C), and an increased duration of elemental responses (Figure 2D), essentially reverting the changes produced by the acute paradigm. Interestingly, the responses of D2-MSNs adapted in a unique way, also increasing basal cAMP levels (Figure S2B) and maximal dopamine-elicited responses (Figures 2B and 2C), but they maintained the same kinetic characteristics seen in mice exposed to a single dose (Figure 2D). We further benchmarked the tolerance paradigm by comparing these results with naive animals (Figures S2C and S2D). This revealed cell-specific adaptations in dopamine processing where tolerance reduced the response amplitude selectively in D1-MSNs (Figure S2E) while shortening the signal duration in both D1- and D2-MSNs (Figure S2F).

We proceeded to analyze signal integration properties of the circuit by delivering trains of stimulation. Consistent with the reversal of the elemental response properties of D1-MSNs toward their drug-naive state, we found that responses from chronically treated mice also partially regained their ability to sum the response into a tetanic wave at 3-min ISIs (Figure 2E). However, at higher stimulation frequencies, the response was virtually indistinguishable from acutely treated mice. A similar normalization of response integration was also seen in D2-MSNs (Figure 2E). Strikingly, the integrated response of chronically exposed D2-MSNs showed a unique feature not observed upon acute stimulation: a prominent rebound wave in the positive direction before returning to a stable basal level (Figures 2E and S2G). Overall, these data suggest that chronic opioid exposure readjusts the circuit properties, diminishing some adaptations produced by the acute exposure, yet maintaining and exacerbating others in a population-specific manner.

We further examined how chronic opioid exposure changes the response properties relative to the drug-naive state, when animals are withdrawn from the drug exposure for 24 h prior to experimentation, modeling an abstinent state (Figure 2A). In this paradigm, we also observed significant changes in the basal cAMP content relative to drug-naive mice: a reduction in D1-MSNs and nearly doubling in D2-MSNs (Figure S2H). Elemental responses of D1-MSNs from opioid-abstinent mice were markedly affected, with a 4-fold decrease in a maximal amplitude and a significant reduction in duration, when compared to drug-naive mice (Figures 2F–2H). Interestingly, the response properties of D2-MSNs remained largely unaffected, with only slightly augmented response amplitude (Figures 2F–2H). These changes were paralleled by massive deficits in the response summation exhibited by D1-MSNs (Figures 2I and S2I), which essentially failed to produce any surge in cAMP at either low or high stimulation frequencies. Again, the response properties of D2-MSNs to the stimulus trains were not significantly different from the drug-naive state (Figure 2I). These observations indicate that opioid abstinence suppresses D1-MSN signaling while sustaining output from D2-MSNs.

### Opioid-Induced Changes in Dopaminergic Responses of MSNs Are Differentially Impacted by Pre- and Postsynaptic Mechanisms in a Neuronal-Type Selective Manner

**Figure 3.**
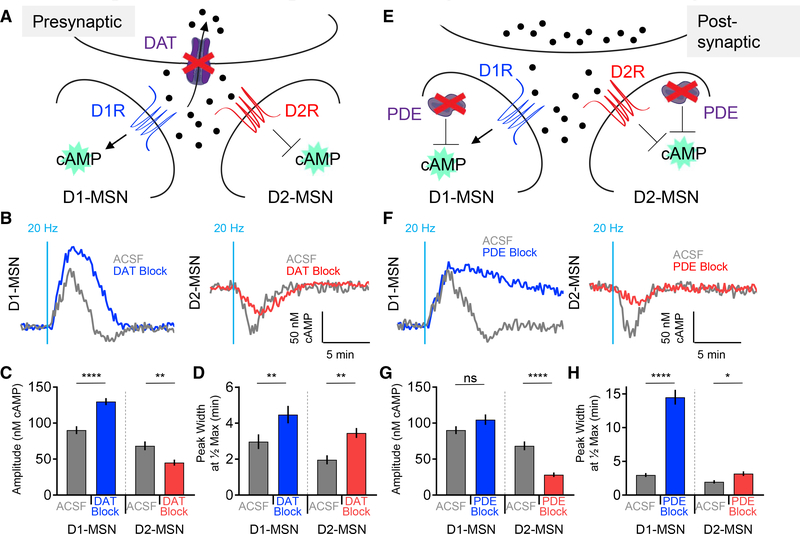
Dopamine Transporter and Phosphodiesterases Regulate Magnitude and Duration of cAMP Signaling (A) DAT inhibition schematic. (B) cAMP response from optical stimulation. (C) Max amplitude cAMP change. D1-MSN ACSF 90.3 ± 5.5 nM cAMP (n = 51 neurons; 7 mice) versus D1-MSN DAT block 139.9 ± 4.8 nM cAMP (n = 38 neurons; 4 mice); t test p < 0.0001, KS D = 0.4892. D2-MSN ACSF 68.5 ± 6.1 nM cAMP (n = 47 neurons; 6 mice) versus D2-MSN DAT block 45.0 ± 4.2 nM cAMP (n = 49 neurons; 4 mice); t test p = 0.0030, KS D = 0.3678. (D) Response duration. D1-MSN ACSF 3.0 ± 0.29 min (n = 51 neurons; 7 mice) versus D1-MSN DAT block 4.5 ± 0.49 min (n = 38 neurons; 4 mice); t test p = 0.0052, KS D = 0.3700. D2-MSN ACSF 2.0 ± 0.25 min (n = 47 neurons; 6 mice) versus D2-MSN DAT block 3.5 ± 0.28 min (n = 49 neurons; 4 mice); t test p = 0.0010, KS D = 0.3969. (E) PDE inhibition schematic. (F) cAMP response from optical stimulation. (G) Max amplitude cAMP change. D1-MSN ACSF 90.3 ± 5.5 nM cAMP (n = 51 neurons; 7 mice) versus D1-MSN PDE block 104.8 ± 7.2 nM cAMP (n = 33 neurons; 4 mice); t test p = 0.2101, KS D = 0.2371. D2-MSN ACSF 68.5 ± 6.1 nM cAMP (n = 47 neurons; 6 mice) versus D2-MSN PDE block 28.3 ± 3.1 nM cAMP (n = 44 neurons; 4 mice); t test p < 0.0001, KS D = 0.5629. (H) Response duration. D1-MSN ACSF 3.0 ± 0.29 min (n = 51 neuron; 7 mice) versus D1-MSN PDE block 14.5 ± 1.10 min (n = 33 neurons; 4 mice); t test p < 0.0001, KS D = 0.8717. D2-MSN ACSF 2.0 ± 0.25 min (n = 47 neurons; 6 mice) versus D2-MSN PDE block 3.2 ± 0.34 min (n = 44 neurons; 4 mice); t test p = 0.0307, KS D = 0.3032.

The profound influence of opioids on dopaminergic signaling in MSNs prompted an investigation of molecular determinants that shape response properties underlying plastic changes. Dopamine is released in the striatum as a diffuse volume transmitter (Liu et al., 2018; Rice and Cragg, 2008; Surmeier et al., 2010), with reuptake into presynaptic terminals by the dopamine transporter (DAT) that controls the extent of dopamine availability (Giros et al., 1996; Jones et al., 1998). Thus, we first examined the contribution of the DAT to cAMP response dynamics by pharmacologically blocking the DAT in acute slices by including a saturating dose of cocaine in the artificial cerebrospinal fluid (ACSF) solution (Figure 3A). We observed that inhibition of the DAT by cocaine, prior to optical stimulation, rapidly induced cAMP changes in both MSN subpopulations (Figures S3A and S3B). This effect plateaued after approximately 30 min, at which point elemental responses to the 20-Hz optical stimulation were then recorded in the presence of DAT inhibition by cocaine. We found that DAT inhibition had pronounced effects on dopamine-elicited responses in both MSN populations (Figure 3B). D1-MSNs displayed a significantly greater response amplitude when the DAT was blocked, compared with the ACSF buffer alone (Figures 3B and 3C). In contrast, D2-MSN responses were significantly diminished by the DAT blockade (Figures 3B and 3C). In addition, DAT inhibition significantly prolonged the response duration in both MSN populations (Figures 3B and 3D).

To probe the contribution of postsynaptic elements, we focused on phosphodiesterases (PDEs), a family of enzymes that metabolize cAMP to delimit signal propagation (Figure 3E). Bath application of a PDE inhibitor, *3-isobutyl-1-methylxanthine* (IBMX), increased the baseline cAMP level in both MSN subpopulations in a dose-dependent manner (Figure S3C). After finding that saturation with IBMX achieved an elevated cAMP baseline, we then optically stimulated the dopamine release. Interestingly, the PDE blockade did not alter the amplitude of the D1-MSN elemental responses, but drastically suppressed the responses of the D2-MSNs (Figures 3F and 3G). PDE inhibition also significantly prolonged the response durations in both MSN populations (Figures 3F and 3H). In summary, we conclude that both presynaptic and postsynaptic elements contribute to distinct phases of dopaminergic responses in discrete populations of MSNs: both the DAT and PDEs regulate response kinetics in both D1-MSNs and D2-MSNs, whereas the response amplitude in D1-MSNs is selectively set by the DAT, but not PDEs.

**Figure 4.**
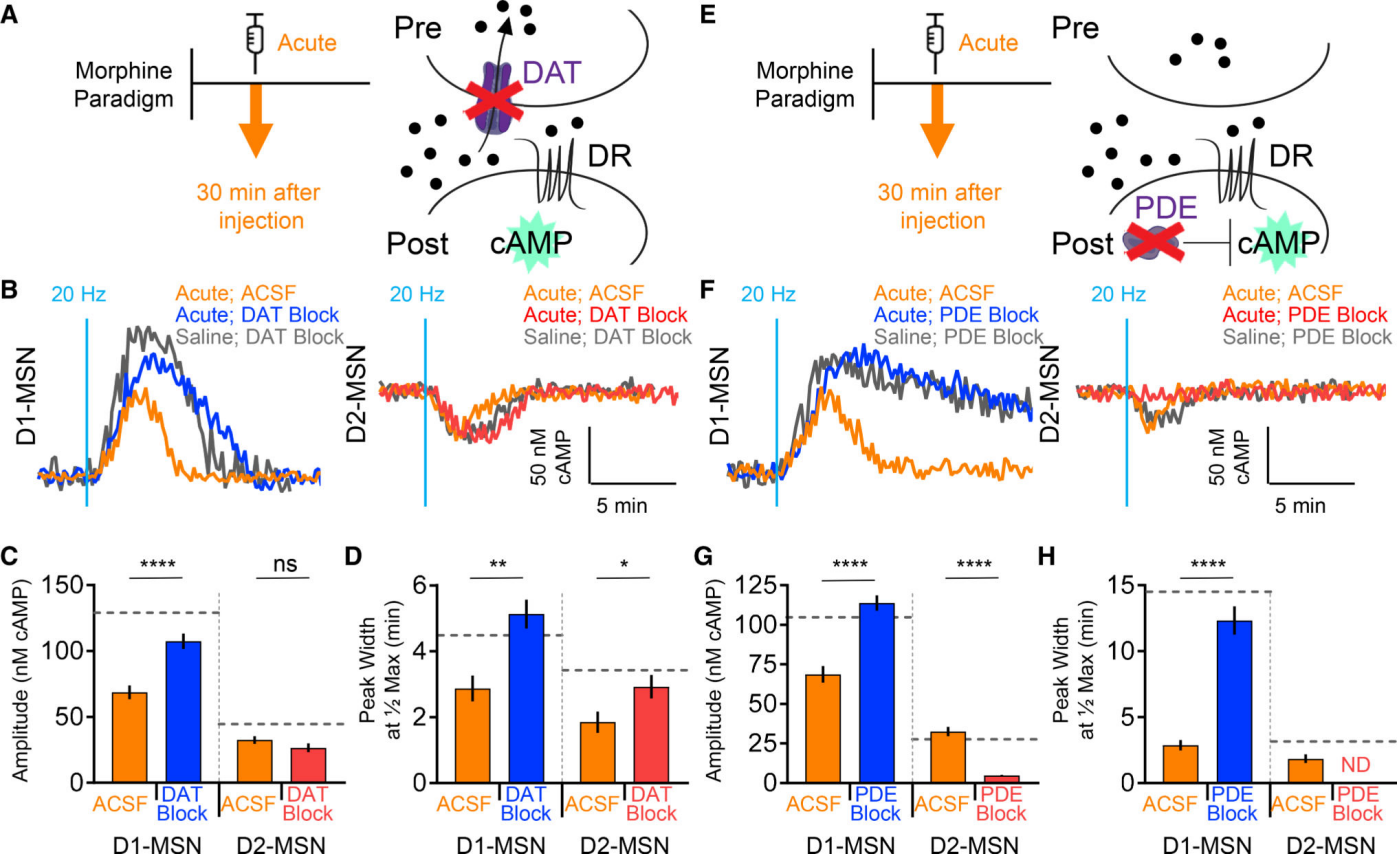
Acute Morphine Modulates DAT and PDE Regulation of cAMP Signaling (A) DAT inhibition schematic. (B) cAMP response from optical stimulation. (C) Max amplitude cAMP change; gray line indicates mean Naive (Saline) DAT Block. D1-MSN ACSF 68.7 ± 5.2 nM cAMP (n = 41 neurons; 6 mice) versus D1-MSN DAT Block 107.4 ± 5.8 nM cAMP (n = 36 neurons; 4 mice); t test p < 0.0001, KS D = 0.5549. D2-MSN ACSF 32.5 ± 2.9 nM cAMP (n = 40 neurons; 5 mice) versus D2-MSN DAT Block 26.6 ± 3.3 nM cAMP (n = 42 neurons; 4 mice); t test p = 0.0766, KS D = 0.2821. (D) Response duration; gray line indicates mean naive (saline) DAT block. D1-MSN ACSF 2.9 ± 0.40 min (n = 41 neurons; 6 mice) versus D1-MSN DAT block 5.1 ± 0.44 min (n = 36 neurons; 4 mice); t test p = 0.0014, KS D = 0.4356. D2-MSN ACSF 1.9 ± 0.32 min (n = 40 neurons; 5 mice) versus D2-MSN DAT block 2.9 ± 0.36 min (n = 42 neurons; 4 mice); t test p = 0.0383, KS D = 0.3107. (E) PDE inhibition schematic. (F) cAMP response from optical stimulation. (G) Max amplitude cAMP change; gray line indicates mean naive (saline) PDE block. D1-MSN ACSF 68.7 ± 5.2 nM cAMP (n = 41 neurons; 6 mice) versus D1-MSN PDE block 113.8 ± 4.8 nM cAMP (n = 38 neurons; 4 mice); t test p < 0.0001, KS D = 0.6733. D2-MSN ACSF 32.5 ± 2.9 nM cAMP (n = 40 neurons; 5 mice) versus D2-MSN PDE block 4.9 ± 0.39 nM cAMP (n = 45 neurons; 4 mice); t test p < 0.0001, KS D = 0.8778. (H) Response duration; gray line indicates mean naive (saline) PDE block. D1-MSN ACSF 2.9 ± 0.40 min (n = 41 neurons; 6 mice) versus D1-MSN PDE block 12.3 ± 1.08 min (n = 38 neurons; 4 mice); t test p < 0.0001, KS D = 0.7015. D2-MSN ACSF 1.9 ± 0.32 min (n = 40 neurons; 5 mice) versus D2-MSN PDE block width, not detected (n = 45 neurons; 4 mice).

Having established the roles of both the DAT and PDEs in shaping dopaminergic signaling, we next sought to determine their contributions to the adaptations in response parameters triggered by opioid exposure. For these studies, we chose to use the acute morphine protocol to minimize compounding the influence of multi-drug interactions, which could trigger neuroplastic events associated with chronic paradigms (Di Chiara and Imperato, 1988; Liang et al., 2016). As with the drug-naive animals, the DAT blockade by cocaine following opioid exposure (Figure 4A) augmented both the amplitudes and durations of the elemental responses induced by the 20-Hz stimulation in the D1-MSNs (Figures 4B–4D). Similarly, DAT inhibition also extended the duration of signaling in D2-MSNs; however, in this population, we observed no significant change in the response amplitude (Figures 4C and 4D).

We next applied a similar strategy to dissect the contribution of postsynaptic elements in opioid influence by blocking the PDE with IBMX (Figure 4E). We found that in opioid-exposed mice, the D2-MSN population exhibited a similar loss of responsiveness upon the PDE blockade as was seen in the naive animals (Figures 4F–4H). The D1-MSN responses of opioidexposed mice were similarly prolonged in duration upon PDE inhibition as were the corresponding responses seen in naive mice. However, the PDE blockade in mice that received morphine produced unique augmentation in the amplitude of D1-MSNs (Figures 4F and 4G). These results suggest that modulation of the dopamine response amplitude by opioids in D1-MSNs involves postsynaptic PDEs and not a presynaptic DAT, whereas in D2-MSNs, amplitude modulation instead involves DAT action and not PDEs.

### Opioids Induce the Plasticity in Stimulus Frequency Tuning and Sensitivity of Dopaminergic Responses in Striatal MSNs

**Figure 5.**
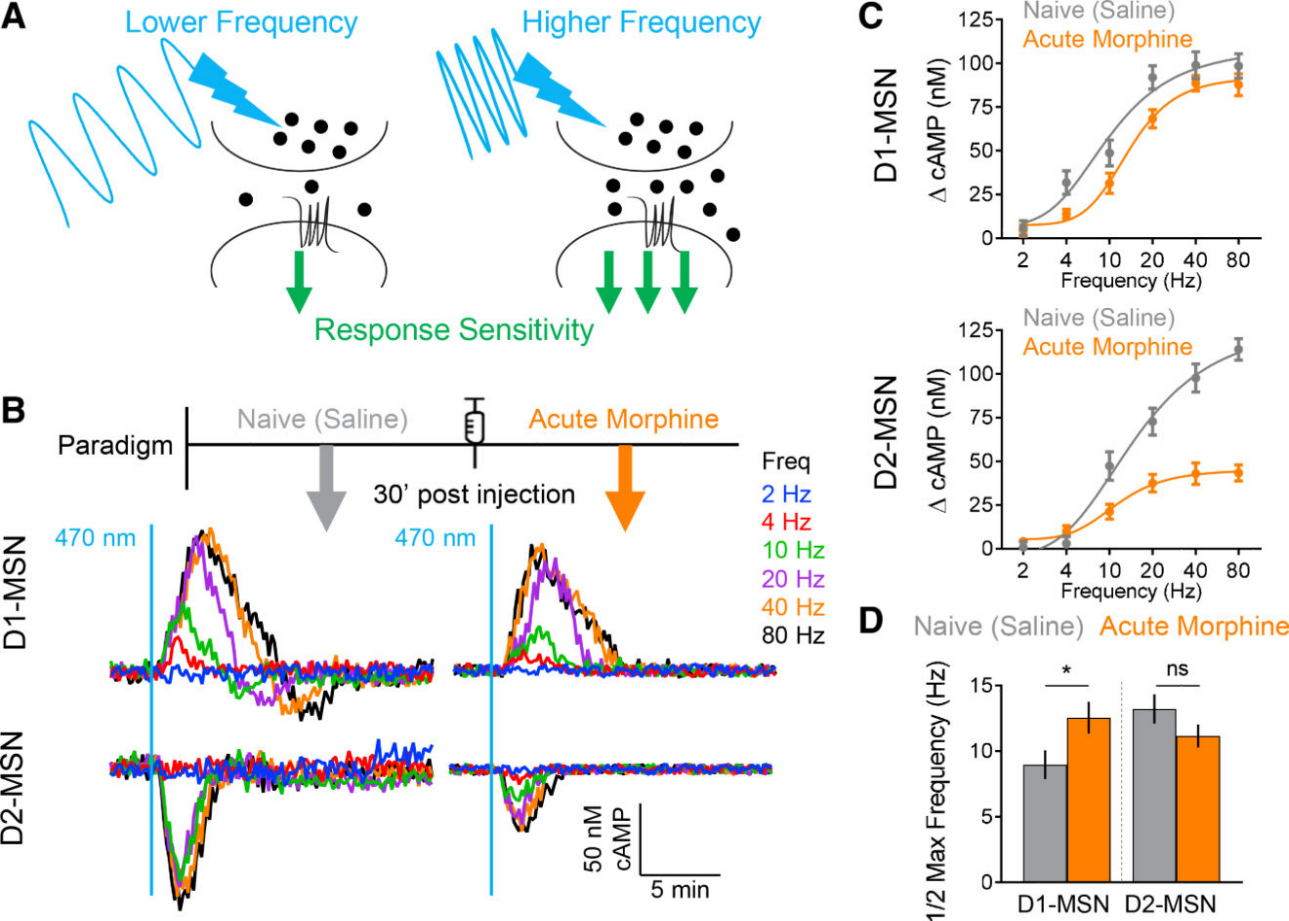
Acute Morphine Modulates Frequency Tuning of Dopaminergic Responses (A) Experimental schematic. (B) cAMP responses varying frequencies of optical stimulation. (C) Max amplitude cAMP change. (D) Stimulation frequency that generated half of the max amplitude change. D1-MSN saline 8.76 ± 1.09 Hz versus D1-MSN morphine 12.56 ± 1.22 Hz; t test p = 0.0421. D2-MSN saline 13.22 ± 1.12 Hz versus D2-MSN morphine 11.16 ± 0.88 Hz; t test p = 0.1628.

To better understand the implications of observed neuron-type-specific alterations on the processing of dopaminergic inputs, we next modeled changes in strength of phasic dopamine release by varying the intensity of the stimulation, while collecting MSN responses over the wide range of the VTA firing frequencies (Figure 5A). Analysis of the stimulus intensity/response relationships revealed two cardinal features impacted by acute opioid influence (Figure 5B). First, morphine exposure selectively reduced D2-MSN signaling capacity by nearly 3-fold without affecting the magnitude of the response in D1-MSNs (Figure 5C). Second, morphine treatment further produced cell-selective effects on the sensitivity to dopaminergic stimulation, causing desensitization of D1-MSNs without significant effects on D2-MSNs (Figures 5C and 5D). We noted that the D1-MSN population exhibited a striking biphasic pattern of the response to dopamine, where an initial surge in cAMP was followed by an oscillatory rebound wave, depressing the signal in the negative direction (Figures 5B and S4A). This rebound cAMP signaling exhibited similar kinetics as the initial wave, and it increased in amplitude with higher frequencies of stimulation (Figures S4B and S4C). Furthermore, the amplitude of the rebound scaled proportionately with the amplitude of the main response component (Figure S4D), thereby limiting the overall dopamine responsiveness of D1-MSNs. Interestingly, morphine exposure completely suppressed this response component (Figure 5B), suggesting that its elimination following morphine exposure likely contributes to elevating baseline cAMP levels. We further noted that the effect of morphine on suppressing the response duration persisted across the entire range of stimulation frequencies in both D2-MSNs and D1-MSNs (Figures S4E and S4F).

**Figure 6.**
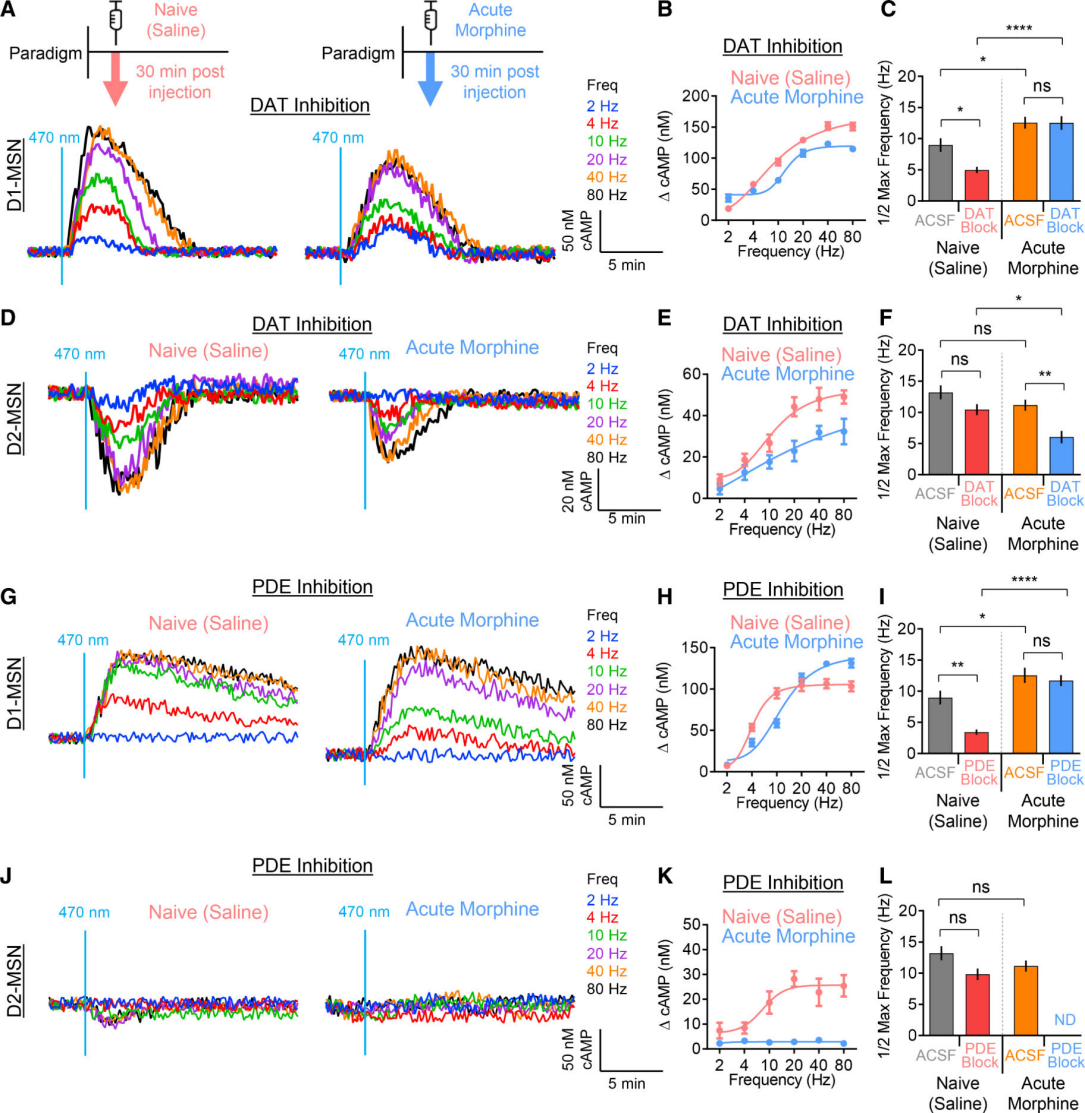
Opioid-Induced Plasticity Modulates Frequency Tuning through the DAT and PDEs (A) D1-MSN response to optical stimulation during DAT inhibition. (B) Max amplitude cAMP change in D1-MSN. (C) Stimulation frequency that generated half of the max amplitude change. D1-MSN naive ACSF 8.76 ± 1.09 Hz, D1-MSN naive DAT block 4.97 ± 0.49, D1-MSN opioid ACSF 12.56 ± 1.22 Hz, D1-MSN opioid DAT block 12.53 ± 1.09 Hz. (D) D2-MSN response to optical stimulation during DAT inhibition. (E) Max amplitude cAMP change in D2-MSN. (F) Stimulation frequency that generated half of the max amplitude change. D2-MSN naive ACSF 13.22 ± 1.12 Hz, D2-MSN naive DAT block 10.45 ± 0.87, D2-MSN opioid ACSF 11.16 ± 0.88 Hz, D2-MSN opioid DAT block 6.05 ± 0.98 Hz. (G) D1-MSN response to optical stimulation during PDE inhibition. (H) Max amplitude cAMP change in D1-MSN. (I) Stimulation frequency that generated half of the max amplitude change. D1-MSN naive ACSF 8.76 ± 1.09 Hz, D1-MSN naive PDE block 3.43 ± 0.42, D1-MSN opioid ACSF 12.56 ± 1.22 Hz, D1-MSN opioid PDE block 11.71 ± 0.88 Hz. (J) D2-MSN response to optical stimulation during PDE inhibition. (K) Max amplitude cAMP change in D2-MSN. (L) Stimulation frequency that generated half of the max amplitude change. D2-MSN naive ACSF 13.22 ± 1.12 Hz, D2-MSN naive PDE block 9.84 ± 0.92, D2-MSN opioid ACSF 11.16 ± 0.88 Hz, D2-MSN opioid PDE block, not detected. Two-way ANOVA; ns p > 0.05, *p < 0.05, **p < 0.01, ***p < 0.001, ****p < 0.0001.

We next interrogated the contributions of the DAT and PDEs to morphine-induced adaptations of frequency tuning and response sensitivity. First, we compared elemental responses evoked by varying frequencies recorded in slices from saline-(naive) and acute morphine-exposed mice following the DAT blockade (Figures 6A–6C). In drug-naive (saline) D1-MSNs, DAT inhibition significantly increased the sensitivity to dopaminergic inputs; however, during acute morphine exposure, this effect on sensitivity was absent (Figures 6B and 6C). During the DAT blockade, morphine still caused a reduction in dopamine sensitivity in D1-MSNs; however, this effect was substantially exacerbated relative to slices without DAT inhibition (Figure 6C). Moreover, DAT inhibition increased the maximum signaling capacity (i.e., response amplitude at max stimulation frequency), and this effect was subdued by acute morphine exposure (Figure S5A). In contrast, in D2-MSNs, DAT inhibition did not affect dopamine sensitivity in naive (saline) mice, but markedly increased it in acute morphine-exposed animals (Figures 6D–6F). Furthermore, DAT inhibition in D2-MSNs significantly reduced the signaling capacity in naive (saline) but not in acute morphine-treated mice (Figure S5B). These findings suggest that dopamine reuptake selectively limits the sensitivity of D1-MSNs to dopaminergic stimulation, whereas acute morphine exposure pivots the role of DAT toward limiting the sensitivity of D2-MSNs to dopamine.

Using similar experimental logic, we next interrogated the contribution of PDEs to morphine-induced changes in frequency tuning of the sensitivity to dopaminergic inputs. In drug-naive (saline) D1-MSNs, blockade of PDEs significantly increased sensitivity to dopamine; however, this effect was not observed in mice exposed to acute morphine (Figures 6G–6I). Again, we observed that the PDE blockade substantially exacerbated the degree of acute morphine-induced modulation of dopamine sensitivity of the D1-MSNs. Surprisingly, PDE did not play a role in D1-MSN signaling capacity in naive (saline) mice; however, following acute morphine treatment, the PDE blockade significantly increased max capacity (Figure S5C). Similar to the DAT blockade in D2-MSNs, inhibition of PDEs in these neurons did not change the sensitivity to dopamine in naive mice (Figures 6J–6L). Strikingly, PDE inhibition completely ablated the responsiveness of D2-MSNs to dopamine in acute morphine-treated animals. Furthermore, the PDE blockade had a general effect of reducing signaling capacity in D2-MSNs (Figure S5D). Thus, we conclude that in the drug-naive (saline) context, PDEs play a cell-specific role in tuning D1-MSN sensitivity to dopamine, whereas following acute morphine exposure, PDEs no longer have the ability to modulate response sensitivity.

### Allostatic Model for Drug-Induced Adaptation in Processing Dopamine Signals by Striatal Neurons

**Figure 7.**
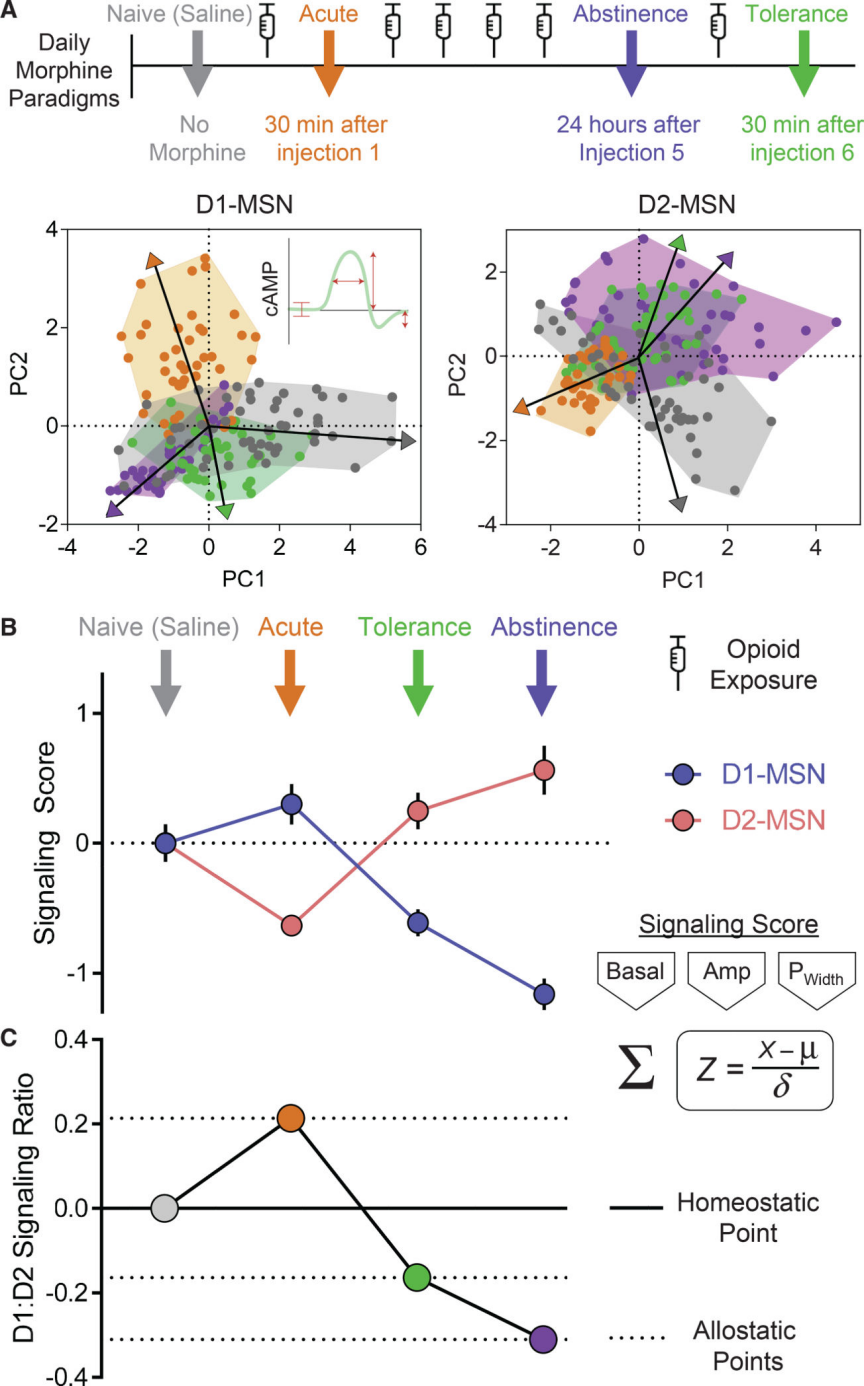
cAMP Signaling Activity between MSN Channels Calculates Shifts in Allostatic States (A) Principle component analysis on correlations utilizing basal cAMP, signal duration, cAMP response amplitude, and cAMP rebound amplitude during phases of opioid exposure. Vectors represent mean population directionality from zero. (B) Aggregate *Z* score of signaling parameters (basal cAMP, response amplitude, and signal duration) from each phase of opioid exposure normalized to naive condition. (C) *Z* score ratio of D1:D2 from each phase of opioid exposure.

Our experiments revealed the extensive impact of opioid exposure on multiple parameters of MSN responses in processing dopaminergic inputs shaped by both pre- and postsynaptic mechanisms. In order to better understand the underlying logic of these changes, we modeled the multivariate data by applying a principle component analysis on correlations to reduce the dimensionality between core signaling features and visualize their impact. We considered changes in key response parameters including the basal cAMP set point, response amplitudes, signaling duration, and oscillatory rebound in defining clusters across morphine treatment paradigms (Figure 7A).

Most notably, this analysis revealed that both types of MSNs, under naive conditions, exhibit broadly dispersed clusters that likely underscore their ability to integrate an adaptive range of neuromodulation and maintain diversity in their responsiveness. Experimental opioid paradigms were characterized by distinct patterns that shared partial overlap with naive neurons, but also clustered in a unique manner. In both MSN subtypes, prolonged opioid exposure in the abstinence and tolerance paradigms sharply shifted principle components in the opposite direction of neurons in mice subjected to the initial acute morphine exposure. The D1-MSN profile from the abstinence and tolerance paradigms concentrated into zones with mutual overlap, yet maintained areas of exclusivity, supporting our observations of overall signaling compression in these neurons (Figure 2). Interestingly, in D2-MSNs, principle components between abstinence and tolerance paradigms shared complete overlaps, thus collapsing to exhibit singular properties. Moreover, in both MSN types, the response parameters of naive neurons clustered in an area at the interface between the divergences observed between the acute and chronic (abstinence/tolerance) paradigms, suggesting that opioids can modulate the mesolimbic circuit to induce bidirectional allostatic plasticity.

To consider directionality of shifts in properties, we further calculated the overall cAMP signaling capacity of MSNs from each opioid paradigm by calculating an aggregate *Z* score, combining key response parameters that defined clusters in the principle component analysis (Figures 7B and S6). This analysis revealed that D1-MSNs exhibited an enhanced signaling in response to acute morphine, whereas the signaling was suppressed during tolerance and abstinence paradigms. Conversely, in the D2-MSN population, acute morphine suppressed signaling, while tolerance and abstinence paradigms profoundly enhanced the signaling score. To further understand the impact of opioids on the coordination of activity between MSN channels, we calculated the ratio of signaling scores between D1- and D2-MSNs (Figure 7C). This revealed a net increase in D1-MSN signaling during acute morphine exposure that reversed to a net increase in D2-MSN signaling during tolerance and abstinence paradigms. Collectively, this reveals the highly plastic nature of the striatal circuit that scales the strength of dopamine responses between neuronal subtypes in a coordinate manner to calculate shifts toward allostatic states. This further underpins the emerging concept whereby opposing MSN channels are concurrently activated to select an appropriate outcome (e.g., reward versus aversion, movement initiation versus suppression) (Cui et al., 2013; Tecuapetla et al., 2014).

## DISCUSSION

### Modulation of Dopamine Signaling to cAMP Provides a Cellular Substrate for Allostatic Adjustments in Addictive States

Continual maintenance of homeostasis is essential for sustaining life in all animals (Cooper, 2008; Koshland, 2002). This requires maintaining stable “pre-set” cellular conditions such as pH, protein levels, and energetics that facilitate configuring higher-level processes required for survival such as core temperature, fluid balance, and neurotransmission (Freddolino and Tavazoie, 2012; Soederberg, 1964). The optimal neutral equilibrium is constantly threatened as organisms cope with changes in the environment (Cannon, 1929). These challenges can result in adaptations that adjust the physiological set point of a given parameter, which then forces stability to be achieved at a novel set point, a process known as allostasis (Sterling and Eyer, 1988). Allostasis is a feed-forward process that deploys resources to actively maintain a non-equilibrium state and support chronic deviations from the homeostatic set point. While being largely an adaptive process, its dysregulation can lead to pathology. This conceptual foundation has been proposed to explain adaptations in the reward circuit during drug addiction (George et al., 2012; Koob and Le Moal, 2001). In this paradigm, the role of central actuator of reward belongs to the neurotransmitter dopamine (Schultz, 2013; Tsai et al., 2009). Drugs with abuse potential, exemplified by opioids in the most extreme case, insult homeostatic balance by interfering with dopaminergic transmission (Di Chiara and Imperato, 1988; Pothos et al., 1991). This altered efficiency of dopamine signaling is thought to play a critical role in programming anticipatory reward calculation (i.e., RPE) (Schultz, 2016), yet the nature of the process that underlies allostatic shifts in the setpoint is not well understood. In this study, we explored the cellular substrates underlying allostatic changes triggered by exposure to opioids, modeling various stages in the development of dependence. We found that cAMP dynamics, as defined by key temporal response patterns to dopamine input frequencies, undergo marked plasticity that impacts the magnitude of signaling strength with a characteristic pattern. Based on these results, we propose that the adjustment of cAMP dynamics in MSNs serves as the key substrate for shifting between allostatic states. We further postulate that these signaling characteristics via cAMP enable the adaptive processing of neuromodulatory inputs, programming homeostatic control as well as physiological and pathological adaptations of its set point.

### Opponent MSN Channels Exhibit Differential Susceptibilities to Opioid-Induced Adaptations

We observed that D1- and D2-MSN subpopulations not only exhibited unique cAMP response profiles to dopamine inputs, but the defining signaling parameters in each neuron type were also distinctly influenced by opioid exposure. In general, an acute opioid exposure enhanced cAMP signaling in D1-MSNs while diminishing it in the D2-MSNs. In contrast, prolonged opioid exposure enhanced signaling in D2-MSNs while suppressing D1-MSN signaling. These observations may provide a molecular basis underlying observations that the acute rewarding effects of morphine may be associated with D1-MSN activity, whereas aversive phenotypes to chronic opioids are thought to be linked to D2-MSNs (Cui et al., 2014b; Kravitz et al., 2012; Tai et al., 2012; Zhu et al., 2016). Our data offer critical support to the idea that MSN subtypes have distinct vulnerabilities to opioids that enable bidirectional processing of dopamine to facilitate dynamic cAMP signaling adjustments, beyond the general involvement of the cAMP system in reward (Nestler, 2016; Terwilliger et al., 1991). For example, it has long been appreciated that the cAMP pathway-mediated activation of the cAMP response-element binding protein (CREB) plays a direct role in morphine reward (Maldonado et al., 1996; Shaw-Lutchman et al., 2002), suggesting a framework for bidirectional modulation of such behavior through cAMP dynamics (Barrot et al., 2002). This is further refined by our observations that acute opioid exposure selectively enhanced basal cAMP in D1-MSNs which are in alignment with an upregulated cAMP pathway (Borgkvist et al., 2007) and exclusive activation of downstream signaling such as DARPP-32 and c-Fos in these neurons in the acute drug exposure setting (Enoksson et al., 2012; Scheggi et al., 2009). Notably, observations that chronic morphine exposure paradigms substantially elevate the baseline cAMP set point selectively in D2-MSNs may underpin the increased excitability of these neurons following drug exposure (Zhu et al., 2016), as well as selective c-Fos induction during opioid withdrawal (Enoksson et al., 2012). Taken together, these observations reinforce the concept of neural population selective shifts in processing neurotransmitter inputs that program addictive states.

### Balance of Activity between MSN Channels Dictates Allostatic Tuning

An output from the basal ganglia that programs behavioral choices is thought to result from coordinated activity between MSN subtypes (Cui et al., 2013; Lobo and Nestler, 2011; Tecuapetla et al., 2014). In the context of addiction, this suggests that drugs of abuse may exert their rewarding effects by triggering allostatic adaptations by impairing the balance between D1- and D2-MSN channels. Consistent with this idea, we found that in naive mice, the cAMP responses in D1- and D2-MSNs are tuned to different frequencies of dopaminergic stimulations. In response to morphine, input tuning to dopamine was selectively altered in the D1-MSNs toward a higher range of stimulation frequencies, reflecting a decrease in sensitivity to dopamine. Since the decoding of reward signals by D1-MSNs occurs by interpreting the phasic patterns of dopamine release that occur at high-frequency stimulation regimens (Schultz, 2013; Schultz et al., 2017; Tsai et al., 2009), this occlusion of responsiveness at low-frequency background dopaminergic tones (Juarez and Han, 2016) could provide a mechanism for priming the D1-MSN pathway for the extraction of reinforcing signals. Interestingly, prolonged opioid exposure triggers an additional mechanism for channel-selective adjustment, switching the circuit bias toward D2-MSNs. We think that these differences in signal integration thresholds between the channels are determined by changes in the temporal resolution of cAMP signals. Illustrating this mechanism, chronic and tolerance paradigms caused a selective loss of the ability of D1-MSNs to resolve discrete cAMP peaks during repeated stimulation, whereas D2-MSNs continued to decode dopamine with robust kinetics. On the basis of these observations, we propose that an allostatic shift in reward valuation upon exposure to opioids is governed by the coordinated changes in the responsiveness of MSN populations to dopamine with initial drug exposure biasing signaling toward D1-MSNs, whereas repeated exposure gradually shifted the balance to favor D2-MSN engagement.

An unexplored area in our study pertains to the modulation of cAMP responses in cholinergic interneurons that also express dopamine receptors, in particular D2R, and that have a documented role in behavioral responses to drugs of abuse (Avena and Rada, 2012). These neurons drive a local dopamine release through the activation of presynaptic nicotinic receptors (nAChRs) on dopaminergic neuron terminals (Cachope et al., 2012; Threlfell et al., 2012), thereby influencing signal processing in MSNs (Mamaligas et al., 2016). Exploring such mechanisms of local microcircuitry in modulating MSN properties will likely be important to address in future studies to fully unravel the allostatic adaptations induced by drugs of abuse.

### Differential Cell-Specific Contributions of Pre- and Postsynaptic Mechanisms to Shaping cAMP Response Characteristics and Plasticity

Since our findings indicate that the plasticity of the dopamine-cAMP signaling axis in MSNs triggers allostatic adaptations induced by opioids, we dedicated significant effort to determining the key factors and molecular players that shape the dynamics of responses to dopamine and uncovered a distinct involvement of both pre- and postsynaptic mechanisms differentially impacting cAMP signaling in D1- and D2-MSNs.

We report that the key role in shaping plasticity of dopamine-cAMP signaling on the pre-synaptic site belongs to the DAT, a molecular player with well-documented involvement in regulating synaptic dopamine concentration (Giros et al., 1996).Strikingly, we observed cell-specific differences in contributions of the DAT to the processing of dopaminergic signals by MSNs. In D1-MSNs, the DAT critically contributed to determining the response magnitude, duration, and sensitivity. In D2-MSNs, the DAT was involved in the modulation of the response magnitude and duration, but not sensitivity. These observations suggest that the normal set point of DAT-mediated reuptake favors D2-MSN signaling and that its reduction may be responsible for shifting the signaling balance toward D1-MSNs. Therefore, inhibiting the DAT by abusive drugs such as cocaine results in reward reinforcement—a phenotype associated with D1-MSN signaling (Calipari et al., 2016). Interestingly, opioid exposure selectively affected DAT-mediated regulation of response properties: it occluded the DAT influence on response sensitivity in D1-MSNs while enabling the DAT to regulate sensitivity but not response magnitude in D2-MSNs. This complex influence of opioids on the interaction between MSN channels is further supported by observations that DAT availability is reduced in chronic opioid users (Liang et al., 2016; Yuan et al., 2017), and yet acute treatment does not reduce the DAT (Simantov, 1993). Thus, it is tempting to speculate that the DAT may assist in pivoting the D1-MSN versus D2-MSN balance in the striatal circuit, contributing toward allostatic tuning of cAMP signaling.

Our studies further reveal selective roles of postsynaptic cAMP-degrading enzymes (PDEs) in influencing dopamine response dynamics and adaptations induced by opioid exposure in a cell-specific manner. PDEs are recognized for their role in striatal pathophysiology (Kelly, 2018) and have been shown to set the basal cAMP tone in striatal neurons (Polito et al., 2015). However, surprisingly little is known about their involvement in dictating response kinetics. We found that PDEs are indispensable for coupling dopamine receptors to cAMP responses in D2-MSNs and thus represent an underappreciated role in decoding external signals in these neurons. Interestingly, we observed no significant influence of PDE activity on the response amplitude of D1-MSNs. In contrast, PDEs substantially influenced the response duration and sensitivity in these neurons. These contributions were drastically changed by opioid exposure, where ensuing plasticity enabled PDEs to modulate the response magnitude while preventing it from controlling the response sensitivity in D1-MSNs, therefore further contributing to altering their response properties. These cell-specific influences may help explain multiple reports of PDE inhibition attenuating D2-MSN-driven phenotypes including morphine-induced withdrawal and conditioned place preference (Hamdy et al., 2001; Itoh et al., 1998; Mamiya et al., 2001; Mu et al., 2014; Núñez et al., 2009).

Collectively, our observations suggest that both presynaptic mechanisms orchestrated by the DAT and postsynaptic properties delineated by PDEs are essential factors that distinctly shape opioid plasticity in the mesolimbic circuit by inducing temporal cell-specific adaptations to bias activity between MSN subtypes. As critical elements in the processing of neurotransmitter responses, both the DAT and PDEs act to selectively adjust the discrete parameters of cAMP signaling to provide both cell-autonomous mechanisms, contributing toward programming diverse allostatic states. Taken together, this prompts a model where opioid exposure is time stamped by distinct profiles of alterations in the processing of dopaminergic signals by striatal neurons, with patterns of bidirectional cAMP modulation serving as substrates for calculating allostatic shifts in reward valuation. We hope this model can provide a useful framework for the exploration of molecular players, circuit mechanisms, and neuromodulatory logic and guide future studies on adaptive neural control of behavior, such as parsing the plastic features of other abusive drugs (e.g., stimulants) or affective disorders (e.g., depression), that are beyond the scope of this study.

## STAR⋆METHODS

### LEAD CONTACT AND MATERIALS AVAILABILITY

Further information and requests for reagents and resources may be directed to, and will be fulfilled by, the Lead Contact, Kirill A. Martemyanov (kirill@scripps.edu). This study did not generate new unique reagents.

### EXPERIMENTAL MODEL AND SUBJECT DETAILS

#### Animal subjects

All experimental work involving mice was approved by The Scripps Research Institute’s IACUC committee in accordance with NIH guidelines. Mice were housed under standard conditions in a pathogen-free facility on a 12:12 light:dark hour cycle with continuous access to food and water. Male and female *CAMPER*^+/+^*:Drd1Cre*^+/−^ and *CAMPER*^+/+^*:Drd2Cre*^+/−^ mice were utilized in these studies and were not subjected to any prior experiments.

### METHOD DETAILS

#### Viral injection

Adult mice were subjected to isoflurane anesthesia for bilateral stereotaxic delivery of AAV5-hSyn-hChR2(H134R)-mCherry (4.1 × 10^12^ GC/ml) through Hamilton syringes. Viral particles were infused at 150 nl/min (1 μL total volume) into the VTA (respective to Bregma, in mm, at a 7° angle: AP = −3.0, ML = ± 1.05, DV −4.6). Sufficient diffusion of viral particles was achieved by allowing the syringes to remain in place for five additional minutes after the infusion was completed.

#### Morphine administration

In opioid paradigms, mice were administered subcutaneous morphine at 15 mg/kg or equivalent volume of saline. The acute paradigm consisted of a single morphine dose followed by slice preparation 30 min post injection. In the abstinence paradigm, mice were administered morphine every 24 h for five consecutive days followed by slice preparation 24 h after the final injection. In the chronic paradigm, mice were administered morphine every 24 h for six consecutive days followed by slice preparation 30 min after the final injection.

#### Acute brain slice preparation

Adult mice (both male and female around 3 months of age) were anesthetized with isoflurane followed by decapitation and rapid excision of the whole brain. In order to maximize preservation of mesolimbic circuit connections, sagittal sections (300 μm) were cut between 15–20° respective to the midline (Wallmichrath and Szabo, 2002) on a vibratome (Leica VT1200S, Germany) in ice cold artificial cerebrospinal fluid (ACSF) equilibrated with 95% O2 and 5% CO2 consisting of (in mM): NaCl (125), KCl (2.5), CaCl_2_ (0.4), MgCl_2_ (1), NaHCO_3_ (25), NaH_2_PO_4_ (1.25), Glucose (25), Kynurenic acid (1). Slices were then transferred to gassed ACSF absent kynurenic acid with 2 mM CaCl_2_ for 30 min prior to performing experiments. Recordings were made in a chamber perfused with gassed ACSF, void of kynurenic acid, at 2 mL/min. Dopamine-mediated responses were isolated by inclusion of picrotoxin (100 mM), DNQX (10 μM), CGP55845 (300 nM), and scopolamine hydrobromide (200 nM) in the ACSF as described (Marcott et al., 2014).

#### Quantitative cAMP imaging

As previously described (Muntean et al., 2018), intracellular cAMP was quantified in realtime *CAMPER* mouse brain slices by imaging FRET changes from NAc MSNs anatomically identified in the ventral region of the striatum approximately 5 mm rostral to midbrain mCherry fluorescence (from VTA hChR infusion). Sub-NAc geography between core and shell was not distinguished. Utilizing a Leica TCS SP8 MP confocal microscope, excitation of FRET donor (mTurquoise) was achieved with a Ti:sapphire laser (Coherent) tuned to 850 nm whereupon simultaneous donor (mTurquoise; 465–505 nm) and acceptor (Venus; 525–600 nm) bandpass emission XYZ image stacks were collected through a 25X objective lens at 10 s intervals. Neuronal cell bodies were defined as regions of interest and raw fluorescence intensity from both channels was used to calculate FRET using ImageJ software. Δ FRET values were converted to cAMP concentrations by interpolation from a standard calibration curve. Channelrhodopsin stimulation in the VTA was achieved with 0.3 mW of 470 nm of light delivered from a 400 μm core fiber optic cannula from a fiber-coupled LED (Thorlabs). Facilitation of optical stimulation trains (20 flashes of light each 2 ms in duration) were configured with a Pulse Train Generator (Prizmatix). DAT blockade was achieved by bath application of cocaine hydrochloride (10 μM). PDE blockade was achieved by bath application of 3-Isobutyl-1-methylxanthine (IBMX) at concentrations indicated in the text.

### QUANTIFICATION AND STATISTICAL ANALYSIS

Statistical analysis was performed using Prism GraphPad software where Student’s t test and ANOVA were used for pairwise comparisons with the use of asterisks to indicate statistical significance (* = p < 0.05, ** = p < 0.01, *** = p < 0.001, **** = p < 0.0001). At least three biological replicates were performed for each experiment with data from all samples included. Graphs report mean values with the presence of error bars to denote standard error of the mean. Principle component analysis on correlations was performed using JMP 14 software.

### DATA AND CODE AVAILABILITY

The datasets supporting the current study are available from the Lead Contact on request.

## Supplementary Material

1

## Figures and Tables

**KEY RESOURCES TABLE:** 

REAGENT or RESOURCE	SOURCE	IDENTIFIER
Bacterial and Virus Strains		
AAV5-hSyn-hChR2(H134R)-mCherry	UNC Vector Core	N/A
Chemicals, Peptides, and Recombinant Proteins		
Morphine sulfate	Sigma-Aldrich	1448005; CAS: 6211–15–0
Kynurenic acid	Sigma-Aldrich	K3375; CAS: 492–27–3
Picrotoxin	Tocris	1128; CAS: 124–87–8
DNQX	Tocris	2312; CAS: 1312992–24–7
CGP55845	Tocris	1248; CAS: 149184–22–5
Scopolamine hydrobromide	Tocris	1414; CAS: 114–49–8
Cocaine hydrochloride	Sigma-Aldrich	1143008; CAS: 53–21–4
IBMX	Tocris	2845; CAS: 28822–58–4
Experimental Models: Organisms/Strains		
Mouse: C57BL/6-Gt(ROSA)26Sortm1(CAG-ECFP*/Rapgef3/Venus*)Kama/J	The Jackson Laboratory	JAX: 032205
Mouse: C57BL/6-Tg(Drd1-cre)EY262Gsat/Mmucd	MMRRC	RRID:MMRRC_017264-UCD
Mouse: C57BL/6-Tg(Drd2-cre)ER43Gsat/Mmucd	MMRRC	RRID:MMRRC_017268-UCD
Software and Algorithms		
ImageJ	https://imagej.nih.gov/ij/	N/A
JMP14	https://www.jmp.com/global-geo-redirects/geohome.html	N/A
GraphPad Prism 6	https://www.graphpad.com/	N/A
Microsoft Office 16	https://www.office.com/	N/A

## Highlights

- Genetically encoded cAMP sensors allow measuring opioid actions in the striatum
- Opioids differentially adjust the strength of medium spiny neuron responses to dopamine
- Synaptic elements shape dopaminergic cAMP plasticity in a cell-type-specific manner
- cAMP serves as a cellular substrate of allostatic changes in dopaminergic signaling

## References

[R1] Avena NM, and Rada PV (2012). Cholinergic modulation of food and drug satiety and withdrawal. Physiol. Behav 706, 332–336. 10.1016/j.physbeh.2012.03.020 PMC4361033 22465312

[R2] Barrot M, Olivier JD, Perrotti LI, DiLeone RJ, Berton O, Eisch AJ, Impey S, Storm DR, Neve RL, Yin JC, (2002). CREB activity in the nucleus accumbens shell controls gating of behavioral responses to emotional stimuli. Proc. Natl. Acad. Sci. USA 99, 11435–11440. 12165570 10.1073/pnas.172091899 PMC123274

[R3] Borgkvist A, Usiello A, Greengard P, and Fisone G (2007). Activation of the cAMP/PKA/DARPP-32 signaling pathway is required for morphine psychomotor stimulation but not for morphine reward. Neuropsychopharmacology 32, 1995–2003. 17251906 10.1038/sj.npp.1301321

[R4] Cachope R, Mateo Y, Mathur BN, Irving J, Wang HL, Morales M, Lovinger DM, and Cheer JF (2012). Selective activation of cholinergic interneurons enhances accumbal phasic dopamine release: setting the tone for reward processing. Cell Rep. 2, 33–41. 22840394 10.1016/j.celrep.2012.05.011 PMC3408582

[R5] Calipari ES, Bagot RC, Purushothaman I, Davidson TJ, Yorgason JT, Peña CJ, Walker DM, Pirpinias ST, Guise KG, Ramakrishnan C, (2016). In vivo imaging identifies temporal signature of D1 and D2 medium spiny neurons in cocaine reward. Proc. Natl. Acad. Sci. USA 113, 2726–2731. 26831103 10.1073/pnas.1521238113 PMC4791010

[R6] Cannon WB (1929). Organization for physiological homeostasis. Physiol. Rev 9, 399–431.

[R7] Carlezon WA Jr., Thome J, Olson VG, Lane-Ladd SB, Brodkin ES, Hiroi N, Duman RS, Neve RL, and Nestler EJ (1998). Regulation of cocaine reward by CREB. Science 282, 2272–2275. 9856954 10.1126/science.282.5397.2272

[R8] Cepeda C, Colwell CS, Itri JN, Chandler SH, and Levine MS (1998). Dopaminergic modulation of NMDA-induced whole cell currents in neostriatal neurons in slices: contribution of calcium conductances. J. Neurophysiol 79, 82–94. 9425179 10.1152/jn.1998.79.1.82

[R9] Cooper SJ (2008). From Claude Bernard to Walter Cannon. Emergence of the concept of homeostasis. Appetite 51, 419–427. 18634840 10.1016/j.appet.2008.06.005

[R10] Covey DP, Roitman MF, and Garris PA (2014). Illicit dopamine transients: reconciling actions of abused drugs. Trends Neurosci. 37, 200–210. 24656971 10.1016/j.tins.2014.02.002 PMC4064368

[R11] Cui G, Jun SB, Jin X, Pham MD, Vogel SS, Lovinger DM, and Costa RM (2013). Concurrent activation of striatal direct and indirect pathways during action initiation. Nature 494, 238–242. 23354054 10.1038/nature11846 PMC4039389

[R12] Cui G, Jun SB, Jin X, Luo G, Pham MD, Lovinger DM, Vogel SS, and Costa RM (2014a). Deep brain optical measurements of cell type-specific neural activity in behaving mice. Nat. Protoc 9, 1213–1228. 24784819 10.1038/nprot.2014.080 PMC4100551

[R13] Cui Y, Ostlund SB, James AS, Park CS, Ge W, Roberts KW, Mittal N, Murphy NP, Cepeda C, Kieffer BL, (2014b). Targeted expression of m-opioid receptors in a subset of striatal direct-pathway neurons restores opiate reward. Nat. Neurosci 17, 254–261. 24413699 10.1038/nn.3622 PMC4008330

[R14] Di Chiara G, and Imperato A (1988). Drugs abused by humans preferentially increase synaptic dopamine concentrations in the mesolimbic system of freely moving rats. Proc. Natl. Acad. Sci. USA 85, 5274–5278. 2899326 10.1073/pnas.85.14.5274 PMC281732

[R15] Donroe JH, and Tetrault JM (2017). Substance Use, Intoxication, and Withdrawal in the Critical Care Setting. Crit. Care Clin 33, 543–558. 28601134 10.1016/j.ccc.2017.03.003

[R16] Enoksson T, Bertran-Gonzalez J, and Christie MJ (2012). Nucleus accumbens D2- and D1-receptor expressing medium spiny neurons are selectively activated by morphine withdrawal and acute morphine, respectively. Neuropharmacology 62, 2463–2471. 22410393 10.1016/j.neuropharm.2012.02.020

[R17] Fan P, Jiang Z, Diamond I, and Yao L (2009). Up-regulation of AGS3 during morphine withdrawal promotes cAMP superactivation via adenylyl cyclase 5 and 7 in rat nucleus accumbens/striatal neurons. Mol. Pharmacol 76, 526–533. 19549762 10.1124/mol.109.057802 PMC2730385

[R18] Freddolino PL, and Tavazoie S (2012). Beyond homeostasis: a predictive-dynamic framework for understanding cellular behavior. Annu. Rev. Cell Dev. Biol 28, 363–384. 22559263 10.1146/annurev-cellbio-092910-154129

[R19] George O, Le Moal M, and Koob GF (2012). Allostasis and addiction: role of the dopamine and corticotropin-releasing factor systems. Physiol. Behav 106, 58–64. 22108506 10.1016/j.physbeh.2011.11.004 PMC3288230

[R20] Gerfen CR (1992). The neostriatal mosaic: multiple levels of compartmental organization in the basal ganglia. Annu. Rev. Neurosci 15, 285–320. 1575444 10.1146/annurev.ne.15.030192.001441

[R21] Gerfen CR, Engber TM, Mahan LC, Susel Z, Chase TN, Monsma FJ Jr., and Sibley DR (1990). D1 and D2 dopamine receptor-regulated gene expression of striatonigral and striatopallidal neurons. Science 250, 1429–1432. 2147780 10.1126/science.2147780

[R22] Giros B, Jaber M, Jones SR, Wightman RM, and Caron MG (1996). Hyperlocomotion and indifference to cocaine and amphetamine in mice lacking the dopamine transporter. Nature 379, 606–612. 8628395 10.1038/379606a0

[R23] Gokce O, Stanley GM, Treutlein B, Neff NF, Camp JG, Malenka RC, Rothwell PE, Fuccillo MV, Südhof TC, and Quake SR (2016). Cellular Taxonomy of the Mouse Striatum as Revealed by Single-Cell RNA-Seq. Cell Rep. 16, 1126–1137. 27425622 10.1016/j.celrep.2016.06.059 PMC5004635

[R24] Gradin VB, Baldacchino A, Balfour D, Matthews K, and Steele JD (2014). Abnormal brain activity during a reward and loss task in opiate-dependent patients receiving methadone maintenance therapy. Neuropsychopharmacology 39, 885–894. 24132052 10.1038/npp.2013.289 PMC3924523

[R25] Graybiel AM (2000). The basal ganglia. Curr. Biol 10, R509–R511. 10899013 10.1016/s0960-9822(00)00593-5

[R26] Hamdy MM, Mamiya T, Noda Y, Sayed M, Assi AA, Gomaa A, Yamada K, and Nabeshima T (2001). A selective phosphodiesterase IV inhibitor, rolipram blocks both withdrawal behavioral manifestations, and c-Fos protein expression in morphine dependent mice. Behav. Brain Res 118, 85–93. 11163637 10.1016/s0166-4328(00)00315-6

[R27] Hikida T, Kimura K, Wada N, Funabiki K, and Nakanishi S (2010). Distinct roles of synaptic transmission in direct and indirect striatal pathways to reward and aversive behavior. Neuron 66, 896–907. 20620875 10.1016/j.neuron.2010.05.011

[R28] Itoh A, Noda Y, Mamiya T, Hasegawa T, and Nabeshima T (1998). A therapeutic strategy to prevent morphine dependence and tolerance by coadministration of cAMP-related reagents with morphine. Methods Find. Exp. Clin. Pharmacol 20, 619–625. 9819808 10.1358/mf.1998.20.7.485728

[R29] Jones SR, Gainetdinov RR, Jaber M, Giros B, Wightman RM, and Caron MG (1998). Profound neuronal plasticity in response to inactivation of the dopamine transporter. Proc. Natl. Acad. Sci. USA 95, 4029–4034. 9520487 10.1073/pnas.95.7.4029 PMC19957

[R30] Josselyn SA, Köhler S, and Frankland PW (2017). Heroes of the Engram. J. Neurosci 37, 4647–4657. 28469009 10.1523/JNEUROSCI.0056-17.2017 PMC6596490

[R31] Juarez B, and Han MH (2016). Diversity of Dopaminergic Neural Circuits in Response to Drug Exposure. Neuropsychopharmacology 41, 2424–2446. 26934955 10.1038/npp.2016.32 PMC4987841

[R32] Kandel ER (2012). The molecular biology of memory: cAMP, PKA, CRE, CREB-1, CREB-2, and CPEB. Mol. Brain 5, 14. 22583753 10.1186/1756-6606-5-14 PMC3514210

[R33] Kelly MP (2018). Cyclic nucleotide signaling changes associated with normal aging and age-related diseases of the brain. Cell. Signal 42, 281–291. 29175000 10.1016/j.cellsig.2017.11.004 PMC5732030

[R34] Koob GF, and Le Moal M (2001). Drug addiction, dysregulation of reward, and allostasis. Neuropsychopharmacology 24, 97–129. 11120394 10.1016/S0893-133X(00)00195-0

[R35] Koshland DE Jr. (2002). Special essay. The seven pillars of life. Science 295, 2215–2216. 11910092 10.1126/science.1068489

[R36] Kravitz AV, Tye LD, and Kreitzer AC (2012). Distinct roles for direct and indirect pathway striatal neurons in reinforcement. Nat. Neurosci 15, 816–818. 22544310 10.1038/nn.3100 PMC3410042

[R37] Liang CS, Ho PS, Yen CH, Yeh YW, Kuo SC, Huang CC, Chen CY, Shih MC, Ma KH, and Huang SY (2016). Reduced striatal dopamine transporter density associated with working memory deficits in opioid-dependent male subjects: a SPECT study. Addict. Biol 21, 196–204. 25439653 10.1111/adb.12203

[R38] Liu C, Kershberg L, Wang J, Schneeberger S, and Kaeser PS (2018). Dopamine Secretion Is Mediated by Sparse Active Zone-like Release Sites. Cell 172, 706–718.e715. 29398114 10.1016/j.cell.2018.01.008 PMC5807134

[R39] Lobo MK, and Nestler EJ (2011). The striatal balancing act in drug addiction: distinct roles of direct and indirect pathway medium spiny neurons. Front. Neuroanat 5, 41. 21811439 10.3389/fnana.2011.00041 PMC3140647

[R40] Madia PA, Dighe SV, Sirohi S, Walker EA, and Yoburn BC (2009). Dosing protocol and analgesic efficacy determine opioid tolerance in the mouse. Psychopharmacology (Berl.) 207, 413–422. 19816677 10.1007/s00213-009-1673-6

[R41] Maldonado R, Blendy JA, Tzavara E, Gass P, Roques BP, Hanoune J, and Schütz G (1996). Reduction of morphine abstinence in mice with a mutation in the gene encoding CREB. Science 273, 657–659. 8662559 10.1126/science.273.5275.657

[R42] Malenka RC, and Kocsis JD (1988). Presynaptic actions of carbachol and adenosine on corticostriatal synaptic transmission studied in vitro. J. Neurosci 8, 3750–3756. 2848109 10.1523/JNEUROSCI.08-10-03750.1988 PMC6569613

[R43] Mamaligas AA, Cai Y, and Ford CP (2016). Nicotinic and opioid receptor regulation of striatal dopamine D2-receptor mediated transmission. Sci. Rep 6, 37834. 27886263 10.1038/srep37834 PMC5122907

[R44] Mamiya T, Noda Y, Ren X, Hamdy M, Furukawa S, Kameyama T, Yamada K, and Nabeshima T (2001). Involvement of cyclic AMP systems in morphine physical dependence in mice: prevention of development of morphine dependence by rolipram, a phosphodiesterase 4 inhibitor. Br. J. Pharmacol 132, 1111–1117. 11226142 10.1038/sj.bjp.0703912 PMC1572651

[R45] Marcott PF, Mamaligas AA, and Ford CP (2014). Phasic dopamine release drives rapid activation of striatal D2-receptors. Neuron 84, 164–176. 25242218 10.1016/j.neuron.2014.08.058 PMC4325987

[R46] Martin-Soelch C, Chevalley AF, Künig G, Missimer J, Magyar S, Mino A, Schultz W, and Leenders KL (2001). Changes in reward-induced brain activation in opiate addicts. Eur. J. Neurosci 14, 1360–1368. 11703464 10.1046/j.0953-816x.2001.01753.x

[R47] Mu Y, Ren Z, Jia J, Gao B, Zheng L, Wang G, Friedman E, and Zhen X (2014). Inhibition of phosphodiesterase10A attenuates morphine-induced conditioned place preference. Mol. Brain 7, 70. 25252626 10.1186/s13041-014-0070-1 PMC4180334

[R48] Muntean BS, Zucca S, MacMullen CM, Dao MT, Johnston C, Iwamoto H, Blakely RD, Davis RL, and Martemyanov KA (2018). Interrogating the Spatiotemporal Landscape of Neuromodulatory GPCR Signaling by Real-Time Imaging of cAMP in Intact Neurons and Circuits. Cell Rep. 22, 255–268. 29298426 10.1016/j.celrep.2017.12.022 PMC5761078

[R49] Nestler EJ (2012). Transcriptional mechanisms of drug addiction. Clin. Psychopharmacol. Neurosci 10, 136–143. 23430970 10.9758/cpn.2012.10.3.136 PMC3569166

[R50] Nestler EJ (2016). Reflections on: “A general role for adaptations in G-Proteins and the cyclic AMP system in mediating the chronic actions of morphine and cocaine on neuronal function”. Brain Res. 1645, 71–74. 26740398 10.1016/j.brainres.2015.12.039 PMC4927417

[R51] Nicola SM, and Malenka RC (1998). Modulation of synaptic transmission by dopamine and norepinephrine in ventral but not dorsal striatum. J. Neurophysiol 79, 1768–1776. 9535946 10.1152/jn.1998.79.4.1768

[R52] Núñez C, González-Cuello A, Sánchez L, Vargas ML, Milanés MV, and Laorden ML (2009). Effects of rolipram and diazepam on the adaptive changes induced by morphine withdrawal in the hypothalamic paraventricular nucleus. Eur. J. Pharmacol 620, 1–8. 19683523 10.1016/j.ejphar.2009.08.002

[R53] Patriarchi T, Cho JR, Merten K, Howe MW, Marley A, Xiong WH, Folk RW, Broussard GJ, Liang R, Jang MJ, (2018). Ultrafast neuronal imaging of dopamine dynamics with designed genetically encoded sensors. Science 360, eaat4422. 29853555 10.1126/science.aat4422 PMC6287765

[R54] Phillips PE, Stuber GD, Heien ML, Wightman RM, and Carelli RM (2003). Subsecond dopamine release promotes cocaine seeking. Nature 422,614–618. 12687000 10.1038/nature01476

[R55] Polito M, Guiot E, Gangarossa G, Longueville S, Doulazmi M, Valjent E, Hervé D, Girault JA, Paupardin-Tritsch D, Castro LR, and Vincent P (2015). Selective Effects of PDE10A Inhibitors on Striatopallidal Neurons Require Phosphatase Inhibition by DARPP-32. eNeuro 2, ENEURO.0060–15.2015. 10.1523/ENEURO.0060-15.2015 PMC4596023 26465004

[R56] Posa L, Accarie A, Noble F, and Marie N (2016). Methadone Reverses Analgesic Tolerance Induced by Morphine Pretreatment. Int. J. Neuropsychopharmacol 19, pyv108. 26390873 10.1093/ijnp/pyv108 PMC4966270

[R57] Pothos E, Rada P, Mark GP, and Hoebel BG (1991). Dopamine microdialysis in the nucleus accumbens during acute and chronic morphine, naloxone-precipitated withdrawal and clonidine treatment. Brain Res. 566, 348–350. 1814554 10.1016/0006-8993(91)91724-f

[R58] Reynolds JN, and Wickens JR (2002). Dopamine-dependent plasticity of corticostriatal synapses. Neural Netw. 15, 507–521. 12371508 10.1016/s0893-6080(02)00045-x

[R59] Rice ME, and Cragg SJ (2008). Dopamine spillover after quantal release: rethinking dopamine transmission in the nigrostriatal pathway. Brain Res. Brain Res. Rev 58, 303–313. 10.1016/j.brainresrev.2008.02.004 PMC2879278 18433875

[R60] Rossetti ZL, Hmaidan Y, and Gessa GL (1992a). Marked inhibition of mesolimbic dopamine release: a common feature of ethanol, morphine, cocaine and amphetamine abstinence in rats. Eur. J. Pharmacol 221, 227–234. 1426002 10.1016/0014-2999(92)90706-a

[R61] Rossetti ZL, Melis F, Carboni S, and Gessa GL (1992b). Dramatic depletion of mesolimbic extracellular dopamine after withdrawal from morphine, alcohol or cocaine: a common neurochemical substrate for drug dependence. Ann. N Y Acad. Sci 654, 513–516. 1632615 10.1111/j.1749-6632.1992.tb26016.x

[R62] Scheggi S, Crociani A, De Montis MG, Tagliamonte A, and Gambarana C (2009). Dopamine D1 receptor-dependent modifications in the dopamine and cAMP-regulated phosphoprotein of Mr 32 kDa phosphorylation pattern in striatal areas of morphine-sensitized rats. Neuroscience 163, 627–639. 19559764 10.1016/j.neuroscience.2009.06.053

[R63] Schultz W (2013). Updating dopamine reward signals. Curr. Opin. Neurobiol 23, 229–238. 23267662 10.1016/j.conb.2012.11.012 PMC3866681

[R64] Schultz W (2016). Dopamine reward prediction-error signalling: a two-component response. Nat. Rev. Neurosci 17, 183–195. 26865020 10.1038/nrn.2015.26 PMC5549862

[R65] Schultz W, Stauffer WR, and Lak A (2017). The phasic dopamine signal maturing: from reward via behavioural activation to formal economic utility. Curr. Opin. Neurobiol 43, 139–148. 28390863 10.1016/j.conb.2017.03.013

[R66] Self DW, Barnhart WJ, Lehman DA, and Nestler EJ (1996). Opposite modulation of cocaine-seeking behavior by D1- and D2-like dopamine receptor agonists. Science 271, 1586–1589. 8599115 10.1126/science.271.5255.1586

[R67] Self DW, Genova LM, Hope BT, Barnhart WJ, Spencer JJ, and Nestler EJ (1998). Involvement of cAMP-dependent protein kinase in the nucleus accumbens in cocaine self-administration and relapse of cocaine-seeking behavior. J. Neurosci 18, 1848–1859. 9465009 10.1523/JNEUROSCI.18-05-01848.1998 PMC6792608

[R68] Shaw-Lutchman TZ, Barrot M, Wallace T, Gilden L, Zachariou V, Impey S, Duman RS, Storm D, and Nestler EJ (2002). Regional and cellular mapping of cAMP response element-mediated transcription during naltrexone-precipitated morphine withdrawal. J. Neurosci 22, 3663–3672. 11978842 10.1523/JNEUROSCI.22-09-03663.2002 PMC6758390

[R69] Shen W, Flajolet M, Greengard P, and Surmeier DJ (2008). Dichotomous dopaminergic control of striatal synaptic plasticity. Science 321, 848–851. 18687967 10.1126/science.1160575 PMC2833421

[R70] Simantov R (1993). Chronic morphine alters dopamine transporter density in the rat brain: possible role in the mechanism of drug addiction. Neurosci. Lett 163, 121–124. 8309616 10.1016/0304-3940(93)90360-w

[R71] Soederberg U (1964). Neurophysiological Aspects of Homeostasis. Annu. Rev. Physiol 26, 271–288. 14145322 10.1146/annurev.ph.26.030164.001415

[R72] Sterling P, and Eyer J (1988). Allostasis: A new paradigm to explain arousal pathology In Handbook of life stress, cognition and health (John Wiley & Sons), pp. 629–649.

[R73] Sun F, Zeng J, Jing M, Zhou J, Feng J, Owen SF, Luo Y, Li F, Wang H, Yamaguchi T, (2018). A Genetically Encoded Fluorescent Sensor Enables Rapid and Specific Detection of Dopamine in Flies, Fish, and Mice. Cell 174, 481–496.e419. 30007419 10.1016/j.cell.2018.06.042 PMC6092020

[R74] Surmeier DJ, Shen W, Day M, Gertler T, Chan S, Tian X, and Plotkin JL (2010). The role of dopamine in modulating the structure and function of striatal circuits. Prog. Brain Res 183, 149–167. 20696319 10.1016/S0079-6123(10)83008-0 PMC4431764

[R75] Svenningsson P, Nairn AC, and Greengard P (2005). DARPP-32 mediates the actions of multiple drugs of abuse. AAPS J. 7, E353–E360. 16353915 10.1208/aapsj070235 PMC2750972

[R76] Tai LH, Lee AM, Benavidez N, Bonci A, and Wilbrecht L (2012). Transient stimulation of distinct subpopulations of striatal neurons mimics changes in action value. Nat. Neurosci 15, 1281–1289. 22902719 10.1038/nn.3188 PMC3951287

[R77] Tecuapetla F, Matias S, Dugue GP, Mainen ZF, and Costa RM (2014). Balanced activity in basal ganglia projection pathways is critical for contraversive movements. Nat. Commun 5, 4315. 25002180 10.1038/ncomms5315 PMC4102112

[R78] Terwilliger RZ, Beitner-Johnson D, Sevarino KA, Crain SM, and Nestler EJ (1991). A general role for adaptations in G-proteins and the cyclic AMP system in mediating the chronic actions of morphine and cocaine on neuronal function. Brain Res. 548, 100–110. 1651140 10.1016/0006-8993(91)91111-d

[R79] Threlfell S, Lalic T, Platt NJ, Jennings KA, Deisseroth K, and Cragg SJ (2012). Striatal dopamine release is triggered by synchronized activity in cholinergic interneurons. Neuron 75, 58–64. 22794260 10.1016/j.neuron.2012.04.038

[R80] Tjon GH, De Vries TJ, Ronken E, Hogenboom F, Wardeh G, Mulder AH, and Schoffelmeer AN (1994). Repeated and chronic morphine administration causes differential long-lasting changes in dopaminergic neurotransmission in rat striatum without changing its delta- and kappa-opioid receptor regulation. Eur. J. Pharmacol 252, 205–212. 7908881 10.1016/0014-2999(94)90598-3

[R81] Tobler PN, Fiorillo CD, and Schultz W (2005). Adaptive coding of reward value by dopamine neurons. Science 307, 1642–1645. 15761155 10.1126/science.1105370

[R82] Tsai HC, Zhang F, Adamantidis A, Stuber GD, Bonci A, de Lecea L, and Deisseroth K (2009). Phasic firing in dopaminergic neurons is sufficient for behavioral conditioning. Science 324, 1080–1084. 19389999 10.1126/science.1168878 PMC5262197

[R83] Volkow ND, Fowler JS, Wang GJ, Baler R, and Telang F (2009). Imaging dopamine’s role in drug abuse and addiction. Neuropharmacology 56 (Suppl 1), 3–8. 18617195 10.1016/j.neuropharm.2008.05.022 PMC2696819

[R84] Volkow ND, Jones EB, Einstein EB, and Wargo EM (2019). Prevention and Treatment of Opioid Misuse and Addiction: A Review. JAMA Psychiatry 76, 208–216. 30516809 10.1001/jamapsychiatry.2018.3126

[R85] Wallmichrath I, and Szabo B (2002). Cannabinoids inhibit striatonigral GABAergic neurotransmission in the mouse. Neuroscience 113, 671–682. 12150787 10.1016/s0306-4522(02)00109-4

[R86] Watts VJ, and Neve KA (2005). Sensitization of adenylate cyclase by Galpha i/o-coupled receptors. Pharmacol. Ther 106, 405–421. 15922020 10.1016/j.pharmthera.2004.12.005

[R87] Yttri EA, and Dudman JT (2016). Opponent and bidirectional control of movement velocity in the basal ganglia. Nature 533, 402–406. 27135927 10.1038/nature17639 PMC4873380

[R88] Yuan J, Liu XD, Han M, Lv RB, Wang YK, Zhang GM, and Li Y (2017). Comparison of striatal dopamine transporter levels in chronic heroin-dependent and methamphetamine-dependent subjects. Addict. Biol 22, 229–234. 26040446 10.1111/adb.12271

[R89] Zheng P, Zhang XX, Bunney BS, and Shi WX (1999). Opposite modulation of cortical N-methyl-D-aspartate receptor-mediated responses by low and high concentrations of dopamine. Neuroscience 91, 527–535. 10366010 10.1016/s0306-4522(98)00604-6

[R90] Zhu Y, Wienecke CF, Nachtrab G, and Chen X (2016). A thalamic input to the nucleus accumbens mediates opiate dependence. Nature 530, 219–222. 26840481 10.1038/nature16954 PMC4814115

