# Supplementary material for: Allostatic Changes in the cAMP System Drive Opioid-Induced Adaptation in Striatal Dopamine Signaling
Source: Cell Rep. Author manuscript; Available in PMC 2019 Nov 21. (PMC6871051; doi:10.1016/j.celrep.2019.09.034)
Supplement: 1 [file NIHMS1542692-supplement-1.pdf]

Cell Reports, Volume *29*

Supplemental Information

Allostatic Changes in the cAMP System Drive Opioid-Induced Adaptation in Striatal Dopamine Signaling

Brian S. Muntean, Maria T. Dao, and Kirill A. Martemyanov

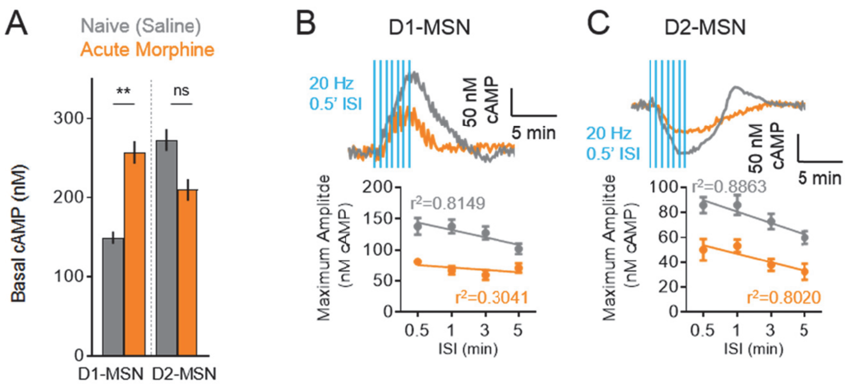

Figure S1. Acute morphine exposure alters kinetic dopamine-mediated signaling parameters in MSNs, Related to Figure 1. A, Baseline cAMP level prior to experimental manipulations in genetically defined striatal subpopulations. Naive D1-MSN 149.4 ± 7.8 nM cAMP (n=51 neurons) vs Acute D1-MSN 272.3 ± 13.9 nM cAMP (n=41 neurons); t-test p<0.0001, Kolmogorov-Smirnov D=0.6585. Naive D2-MSN 256.6 ± 14.1 nM cAMP (n=47 neurons) vs Acute D2-MSN 210.7 ± 12.9 nM cAMP (n=40 neurons); t-test p=0.1521, Kolmogorov-Smirnov D=0.2441. **B,** cAMP responses in D1-MSN from 20 Hz stimulation presented at 0.5 minute inter-stimulation intervals (ISI). Maximum cAMP amplitude from varying ISI protocols were quantified. **C,** cAMP responses in D2-MSN from 20 Hz stimulation presented at 0.5 minute inter-stimulation intervals (ISI). Maximum cAMP amplitude from varying ISI protocols were quantified.

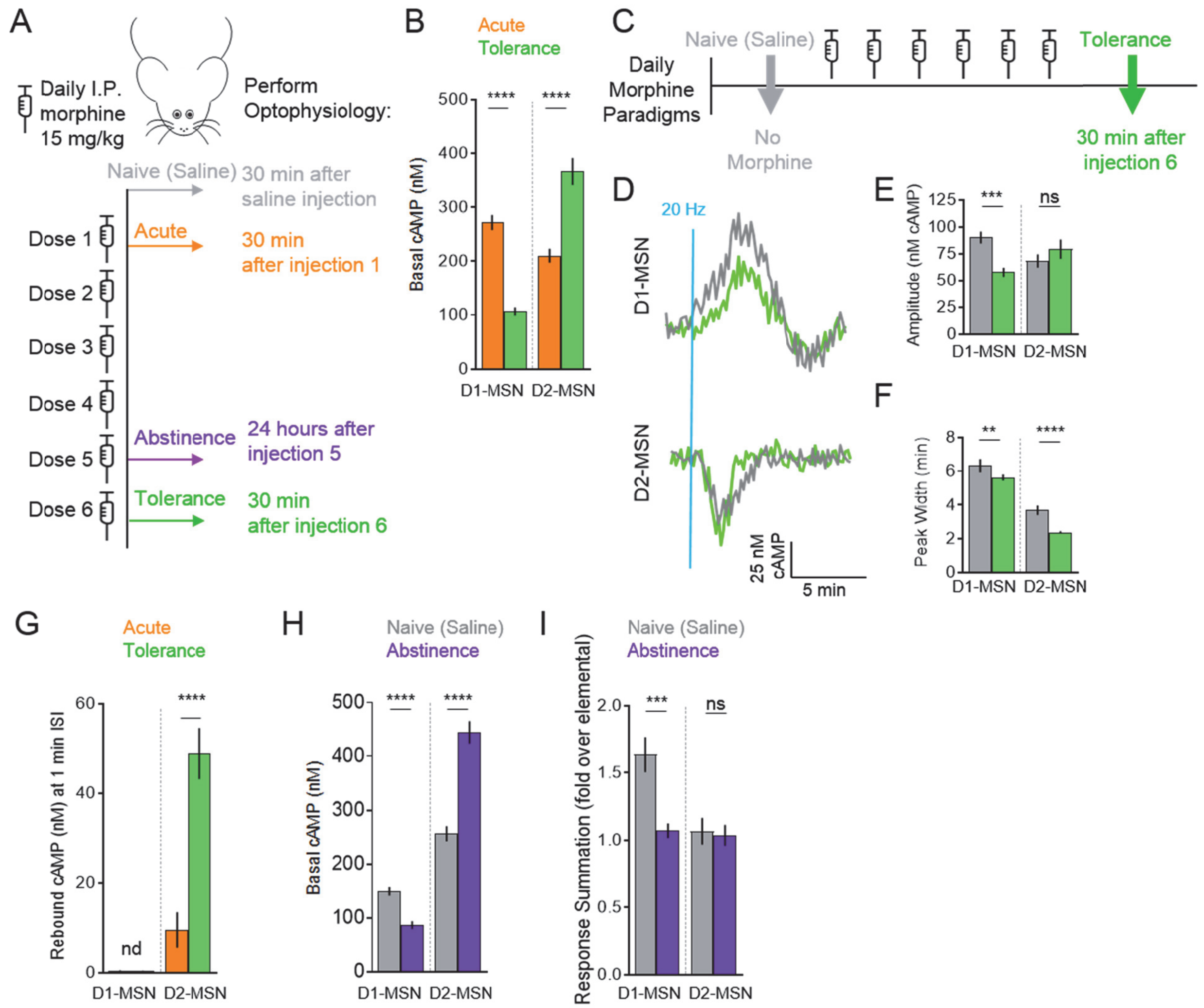

Figure S2. Intrinsic neuroadaptive changes in MSNs following exposure to opioids, Related to Figure 2. A, Schematic of four morphine paradigms utilized. **B,** Baseline cAMP level. Acute D1-MSN 272.3 ± 13.9 nM cAMP (n=41 neurons) vs Tolerance D1-MSN 106.4 ± 7.3 nM cAMP (n=39 neurons); t-test p<0.0001, KS D=0.8049. Acute D2-MSN 210.7 ± 12.9 nM cAMP (n=40 neurons) vs Tolerance D2-MSN 366.6 ± 25.3 nM cAMP (n=46 neurons); t-test p<0.0001, KS D=0.5435. **C,** Experimental design to compare six daily morphine injections (Tolerance) with saline treatment (Naive). **D,** cAMP response from optical stimulation. **E,** Max amplitude cAMP change. Naive D1-MSN 90.3 ± 5.5 nM cAMP (n=51 neurons; 7 mice) vs Tolerance D1-MSN 58 ± 4.5 nM cAMP (n= 39 neurons; 4 mice); t-test p=0.0004, KS D=0.4419. Naive D2-MSN 68.5 ± 6.1 nM cAMP (n=47 neurons; 6 mice) vs Tolerance D2-MSN 80 ± 5.4 nM cAMP (n=46 neurons; 5 mice); t-test p= 0.1053, KS D=0.2516. **F,** Response duration. Naive D1-MSN 6.3 ± 0.39 minutes (n=51 neurons; 7 mice) vs Tolerance D1-MSN 5.6 ± 0.19 minutes (n=39 neurons; 4 mice); t-test p=0.0017, KS D=0.3997. Naive D2-MSN 3.7 ± 0.27 minutes (n=47 neurons; 6 mice) vs Tolerance D2-MSN 2.4 ± 0.07 minutes (n=46 neurons; 5 mice); t-test p<0.0001, KS D=0.5747. **G,** Max amplitude cAMP change in opposite direction of the initial response before returning to baseline. Acute D1-MSN 0.35 ± 0.02 nM cAMP (n=41 neurons) vs Tolerance D1-MSN 0.39 ± 0.03 nM cAMP (n=39 neurons); t-test p=0.0701, KS D=0.2896. Acute D2-MSN 9.6 ± 3.9 nM cAMP (n=40 neurons) vs Tolerance D2-MSN 49.3 ± 5.6 nM cAMP (n=46 neurons); t-test p< 0.0001, KS D=0.9783. **H,** Baseline cAMP level. Naive D1-MSN 149.4 ± 7.8 nM cAMP (n=51 neurons) vs Abstinence D1-MSN 86.6 ± 7.6 nM cAMP (n=35 neurons); t-test p<0.0001, KS D=0.5272. Naive D2-MSN 256.6 ± 14.1 nM cAMP (n=47 neurons) vs Abstinence D2-MSN 444.0 ± 21.2 nM cAMP (n=38 neurons); t-test p<0.0001, KS D=0.692. **I,** Summation of the cAMP response from 3' ISI data calculated by dividing max response by peak response to initial stimulation. Naive D1-MSN 1.63 ± 0.13 fold (n=16 neurons) vs Abstinence D1-MSN 1.07 ± 0.054 fold (n=12 neurons); t-test p=0.0009, KS D=0.75. Naive D2-MSN 1.07 ± 0.099 fold (n=18 neurons) vs Abstinence D2-MSN 1.04 ± 0.079 fold (n=16 neurons); t-test p=0.8558, KS D=0.75.

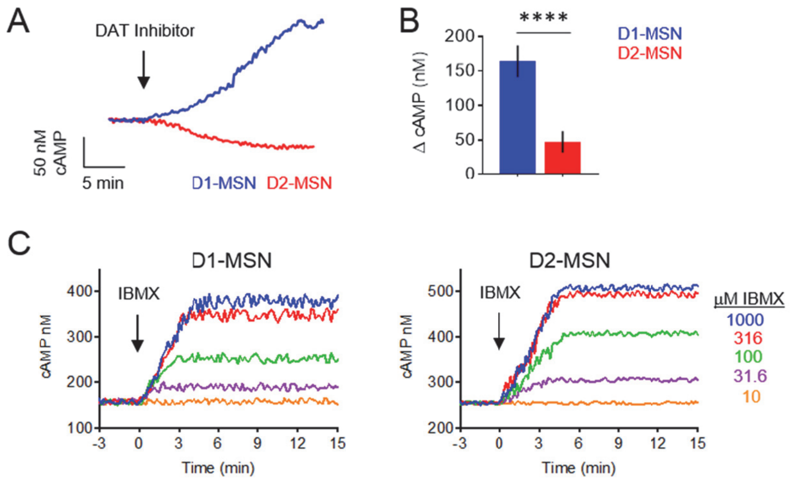

Figure S3. DAT and PDE inhibition influence basal cAMP level in MSNs, Related to Figure 3. A, cAMP response to bath application of DAT inhibitor (cocaine, 10 μM). **B,** Maximum amplitude cAMP change induced by cocaine. D1-MSN 164 ± 23 nM cAMP (n=41 neurons) vs D2-MSN 47 ± 16 nM cAMP (n= 55 neurons); t-test $p<0.0001$. **C,** cAMP response to bath application of nonspecific PDE inhibitor (IBMX).

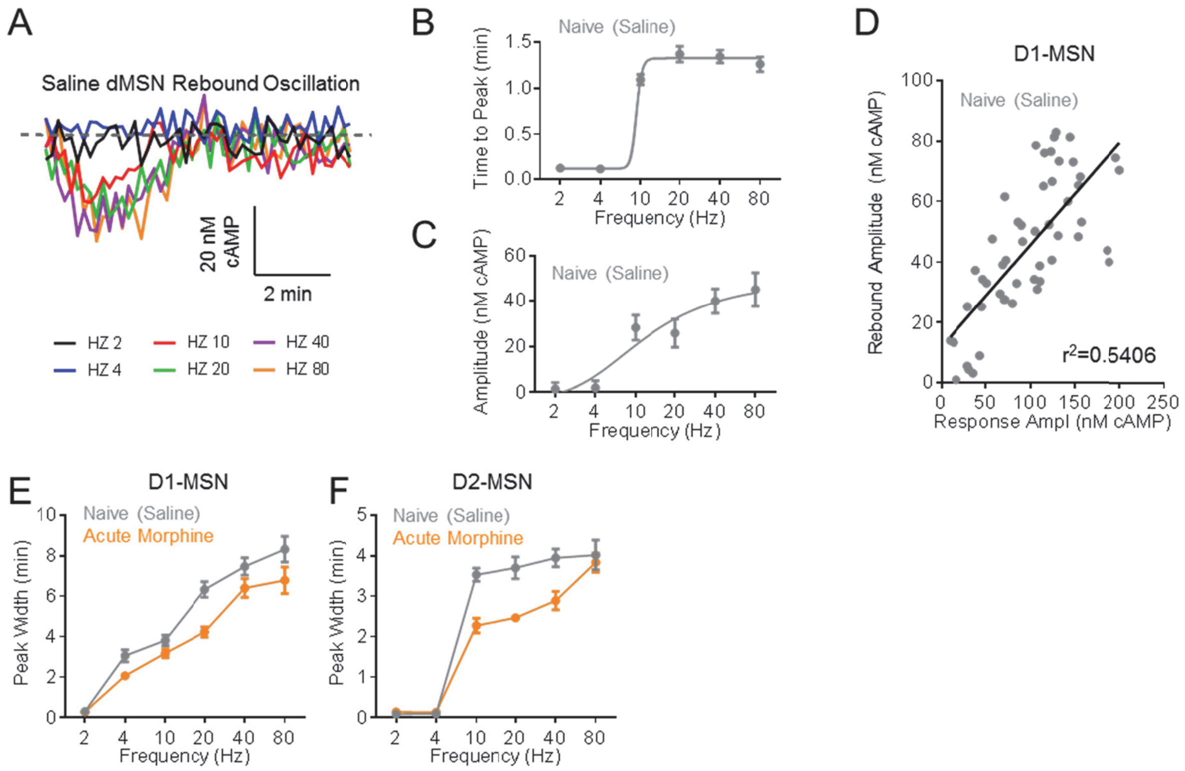

Figure S4. Stimulation frequency influence on cAMP rebound oscillation and response kinetics, Related to Figure 5. A, cAMP change in D1-MSN in the opposite direction of the initial response before returning to baseline. Traces highlight rebound cAMP from data from Fig. 5b. **B,** Time to peak for cAMP rebound in D1-MSN Saline from varying stimulation frequencies. **C,** Maximum cAMP rebound amplitude in D1-MSN Saline from varying stimulation frequencies. **D**, Correlation of cAMP rebound and initial response amplitude in D1-MSN Saline at 80 Hz stimulation. **E,** Peak width of cAMP response from varying stimulation frequencies in D1-MSN. **F,** Peak width of cAMP response from varying stimulation frequencies in D2-MSN.

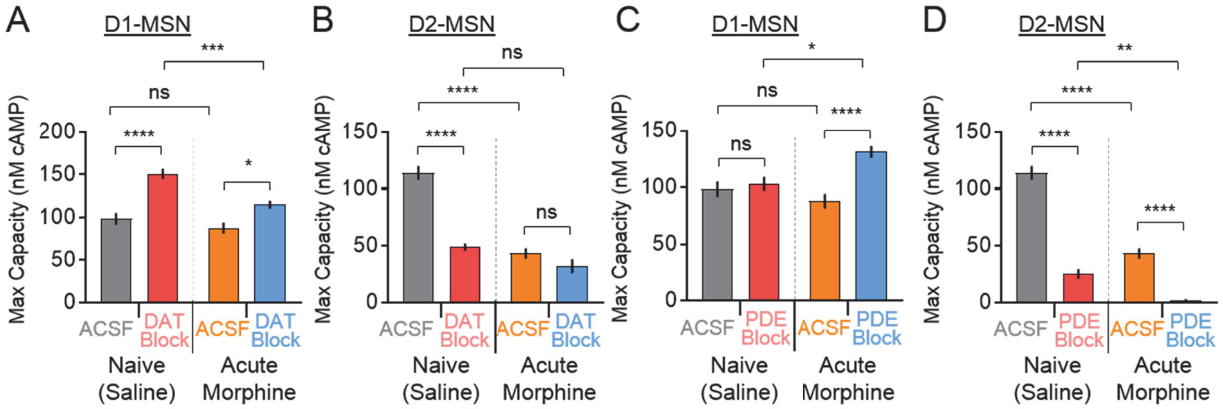

Figure S5. Regulation of maximum signaling capacity by DAT and PDE, Related to Figure 6. Maximum amplitude cAMP change at 80 Hz stimulation frequency. **A,** D1-MSN Naive ACSF 98.44 ± 6.90 nM cAMP, D1-MSN Naive DAT block 151.17 ± 5.85 nM cAMP, D1-MSN Opioid ACSF 87.90 ± 6.25 nM cAMP, D1-MSN Opioid DAT block 114.85 ± 4.37 nM cAMP. **B,** D2-MSN Naive ACSF 113.52 ± 6.15 nM cAMP, D2-MSN Naive DAT block 49.10 ± 3.25 nM cAMP, D2-MSN Opioid ACSF 43.31 ± 4.32 nM cAMP, D2-MSN Opioid DAT block 33.43 ± 5.27 nM cAMP. **C,** D1-MSN Naive ACSF 98.44 ± 6.90 nM cAMP, D1-MSN Naive PDE block 103.14 ± 6.05 nM cAMP, D1-MSN Opioid ACSF 87.90 ± 6.25 nM cAMP, D1-MSN Opioid PDE block 131.08 ± 5.52 nM cAMP. **D,** D2-MSN Naive ACSF 113.52 ± 6.15 nM cAMP, D2-MSN Naive PDE block 24.74 ± 3.50 nM cAMP, D2-MSN Opioid ACSF 43.31 ± 4.32 nM cAMP, D2-MSN Opioid PDE block 2.09 ± 0.15 nM cAMP. Two-way ANOVA; ns $p>0.05$, * $p<0.05$, ** $p<0.01$, *** $p<0.001$, **** $p<0.0001$.

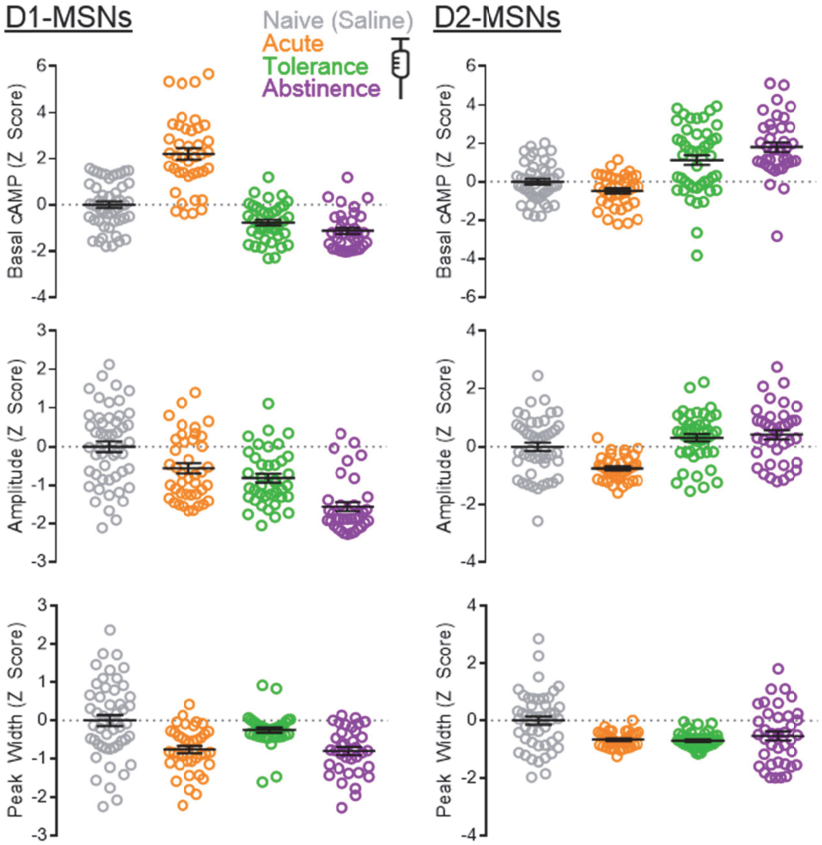

Figure S6. Z score MSN signaling parameters, Related to Figure 7. Individual Z score data normalized to Naive condition for basal cAMP, response amplitude, and peak width in both D1-MSN and D2-MSN.
